# Putative metabolic pathway for the bioproduction of bikaverin and intermediates thereof in the wild *Fusarium oxysporum* LCP531 strain

**DOI:** 10.1186/s13568-019-0912-4

**Published:** 2019-11-20

**Authors:** Juliana Lebeau, Thomas Petit, Laurent Dufossé, Yanis Caro

**Affiliations:** 1 Laboratoire de Chimie des Substances Naturelles et des Sciences des Aliments (LCSNSA), Université de La Réunion, 15 Avenue René Cassin, CS 92003, 97744 Saint-Denis, Réunion France; 2 Département Hygiène Sécurité Environnement (HSE), IUT La Réunion, Université de La Réunion, 40 Avenue de Soweto, BP 373, 97455 Saint-Pierre, Réunion France

**Keywords:** *Fusarium oxysporum*, Naphthoquinones, Bikaverin, Norbikaverin, Beauvericin, Ergosterol

## Abstract

Fungal naphthoquinones, like red bikaverin, are of interest due to their growing applications in designing pharmaceutical products. Though considerable work has been done on the elucidation of bikaverin biosynthesis pathway in *Fusarium fujikuroi,* very few reports are available regarding its bioproduction in *F.* *oxysporum*. We are hereby proposing a putative metabolic pathway for bikaverin bioproduction in a wild *F. oxysporum* strain by cross-linking the pigment profiles we obtained under two different fermentation conditions with literature. Naphthoquinone pigments were extracted with a pressurized liquid extraction method, and characterized by HPLC–DAD and UHPLC-HRMS. The results led to the conclusions that the *F.* *oxysporum* LCP531 strain was able to produce bikaverin and its various intermediates, e.g., pre-bikaverin, oxo-pre-bikaverin, dinor-bikaverin, me-oxo-pre-bikaverin, and nor-bikaverin, in submerged cultures in various proportions. To our knowledge, this is the first report of the isolation of these five bikaverin intermediates from *F.* *oxysporum* cultures, providing us with steady clues for confirming a bikaverin metabolic pathway as well as some of its regulatory patterns in the *F. oxysporum* LCP531 strain, based on the previously reported model in *F. fujikuroi*. Interestingly, norbikaverin accumulated along with bikaverin in mycelial cells when the strain grew on simple carbon and nitrogen sources and additional cofactors. Along bikaverin production, we were able to describe the excretion of the toxin beauvericin as main extrolite exclusively in liquid medium containing complex nitrogen and carbon sources, as well as the isolation of ergosterol derivate in mycelial extracts, which have potential for pharmaceutical uses. Therefore, culture conditions were also concluded to trigger some specific biosynthetic route favoring various metabolites of interest. Such observation is of great significance for selective production of pigments and/or prevention of occurrence of others (*aka* mycotoxins).

## Introduction

Literature is now abundantly reporting the significant application of metabolites from ascomycetous fungi in the industry, through the production of various bioactive compounds, such as plant hormones, enzymes, organic acids, mycotoxins as well as natural pigments (Mapari et al. 2008; Sutthiwong et al. 2013; Dufossé et al. 2014; Caro et al. 2017; Lebeau et al. 2017; Gmoser et al. 2017). In accordance with their genetic potential, *Fusarium* species were found to produce a large panel of secondary metabolites, all diverse in both their structures and biological activities (Pessôa et al. 2017; Abdel-Azeem et al. 2019). Some of these secondary metabolites were characterized as mycotoxins negatively affecting human and animal health. Six main classes have been reported so far and encompass trichothecenes (including deoxynivalenol, nivalenol and T-2 toxin), fumonisins (Proctor et al. 2008), zearalenone (Gaffoor and Trail 2006; Lysøe et al. 2006), beauvericin (Fotso et al. 2002; Zhan et al. 2007), fusaric acid (Bacon et al. 1996; Son et al. 2008; Niehaus et al. 2014) and fusarin C (Wiebe and Bjeldanes 1981; Song et al. 2004; Díaz-Sánchez et al. 2012). Recent studies have described new emerging mycotoxins such as fusaproliferin, enniatins, apicidins, fujikurins, and moniliformin, but with still limited information available on these compounds yet (Cortinovis et al. 2013; Escrivá et al. 2015; Nazari et al. 2015).

*Fusarium* species have also been recognized as promising sources of secondary colored metabolites (e.g., fungal pigments) with potential as positive biological activities in pharmaceutical and medical fields (Pessôa et al. 2017; Caro et al. 2017; Abdel-Azeem et al. 2019; Ramesh et al. 2019). In addition to their structural diversity, fungal pigments were proven as promising bioactive compounds with a wide range of potential applications in various industrial domains, including but not limited to medical, pharmaceutical and agrochemical applications, consequently significantly enlarging their initial use as coloring agents in food and beverages, animal feeds, cosmetics, textile, leather, pulp and paper industries (Dufossé et al. 2014; Gmoser et al. 2017; Caro et al. 2017). Therefore, fungal-originated pigments have been gaining increased interest over the last decade, and nowadays start to find new usages in the development of various antibiotics, immunosuppressants, antitumoral and anti-cancer drugs (Fouillaud et al. 2016; Ramesh et al. 2019; Abdel-Azeem et al. 2019).

Some *Fusarium* species produce bioactive pigments such as carotenoids (Garbayo et al. 2003; Avalos et al. 2007; review in: Avalos et al. 2017) and naphthoquinone pigments (Tatum et al. 1985; Norred et al. 1992; Proctor et al. 2007; review in: Caro et al. 2017). The biosynthesis of naphthoquinone pigments in some *Fusarium* species was shown to be the main response to environmental stresses, observed under conditions of growth inhibition or arrest (Medentsev et al. 2005). Furthermore, the conservation, replacement and development of redundant pigment systems strongly indicated that pigmentation plays a key role in the survival of the members of the *Fusarium* genus. Many *Fusarium* naphthoquinone pigments, like aurofusarin (Kim et al. 2005; Frandsen et al. 2006), fusarubin (Studt et al. 2012) and bikaverin (Brewer et al. 1973; review in: Limón et al. 2010; Lale and Gadre 2016; Lebeau et al. 2019) exhibit useful biological activities. They are recognized as mycotoxins and this fact is important in safety concerns, mainly considering their possible applications in agrochemical, pharmacological and medical sectors (Caro et al. 2017; Abdel-Azeem et al. 2019; Lebeau et al. 2019). For example, red bikaverin is known to possess antitumor activity with potential as pharmaceutical drugs against lymphoma, carcinoma and sarcoma amongst others (Henderson et al. 1977; Zhan et al. 2007; Son et al. 2008; Limón et al. 2010; Nirmaladevi et al. 2014). In terms of industrial applications, some studies describe the use of red pigments produced by *Fusarium* strains in dyeing processes of diverse materials showing the potential of these compounds as alternative dyes in textile industry (Velmurugan et al. 2010). Additionally, bikaverin was also proven as promising source for bio-based blue pigment for use in dyeing of textiles and plastics as recently patented (BR102013015305) (Bicas and Silva 2013). Thus, due to chemical and biological properties of naphthoquinones from *Fusarium* sp., these compounds may be applied not only in medical fields but also as textile and material dyes.

Although considerable work was performed for the bikaverin pathway from *Fusarium* *fujikuroi (*Arndt et al. 2015), very few studies are available regarding its bioproduction in other *Fusarium* species. Indeed, pigment profiles and shades have been widely concluded as versatile from one *Fusarium* sp to another (Caro et al. 2017; Lebeau et al. 2017), suggesting that metabolic pathways and intermediates are likely to be different. In our previous article, two novel wild-type purple naphthoquinone pigments produced by *F. oxysporum* LCP531 were firstly observed along with the constant biosynthesis of bikaverin (Lebeau et al. 2019). Such reports have never been made before, providing strong evidence that other versions of the bikaverin route exist and need to be elucidated. Similar work was performed on alternative routes for carotenoids synthesis in red seaweeds with particular intermediates that are different from the main terrestrial carotenoids pathway model known (Koizumi et al. 2018). Moreover, to perform future rational-design engineering and/or strains optimization for the bioproduction of specific polyketides, better knowledge of the involved metabolic pathways and potential regulatory systems are key elements for success. Therefore, this article focuses on the use of one particular phytopathogenic *Fusarium oxysporum* LCP531 strain to open the way to the determination of the putative metabolic pathway for the bioproduction of bikaverin and its intermediates based on the naphthoquinone pigment profiles of *F. oxysporum* when cultured in submerged conditions. We previously demonstrated that the culture conditions were affecting the pigment profiles of *F. oxysporum* LCP531 (Lebeau et al. 2019), therefore by now elucidating specific metabolic routes of potent pigment for human application (bikaverin), one can easily perceive the potential economic interests that can be drawn.

## Materials and methods

### Submerged fermentation of fungal strain

The strain *Fusarium oxysporum* (collection number LCP 531, soil pathogen sampled on Lucerne host in Indochina) was bought from the fungal culture collection of the Museum d’Histoire Naturelle de Paris (Paris, France). For submerged fermentation, two liquid media were used as culture medium and prepared using sterile distilled water: Potato Dextrose Broth (PDB) and Defined Minimal Dextrose broth (DMD), according to Lebeau et al. (2017) (Additional file 1: Table S1). The pH of the culture medium was adjusted to 6.0 ± 0.2 using 0.1 M HCl prior to sterilization at 121 °C for 15 min. Pre-cultures and cultivations were carried out in 250 mL Erlenmeyer flasks containing 100 mL of sterilized culture medium. The flasks were incubated at 26 °C for 7 days and agitated at 150 rpm using a rotary agitator (Infors Multitron HT).

### Biomass separation and extraction of the fungal secondary metabolites

After 7 days of fermentation, the culture broth and fungal biomass were separated by centrifugation at 10,000 rpm for 10 min (Centrifuge Sigma 3 K 3OH and 19776-H rotor) and vacuum filtration using Whatman™ filter paper GF/C disc (Merck). The mycelial cells and the culture filtrate were frozen (− 84 °C) and then lyophilized (FreeZone 2.5 Liter 50C Benchtop freeze dryer, LABCONCO, Kansas City, MO, USA). Then, the dried biomass was weighed to estimate the mycelial biomass, and was further ground to a fine powder by mechanical grinding before performing pressurized liquid extraction. The fungal secondary metabolites from both the mycelial biomass and the culture filtrate were extracted and fractionated using a six-stage pressurized liquid extraction method according to Lebeau et al. (2017). Samples were transferred to a 10-mL stainless steel extraction cell and pressurized liquid extraction was performed on a Dionex ASE system (ASE™ 350 apparatus, Dionex, Germering, Germany) at 90 °C and 1500 psi. The sequence of solvents was set to display a decreasing polarity profile: purified water was used as the first extraction solvent, followed by 50% methanol, then 50% ethanol, > 99.9% methanol, and MeOH:EtOH (1/1, v/v), and > 99.9% ethanol as the last extraction solvent. Solvents (methanol and ethanol, 99.9%-HPLC quality) were obtained from Carlo Erba (Val de Reuil, France). Purified water was obtained from a Milli-Q system (EMD Millipore Co., Billerica, MA, USA).

The intracellular extracts (IC) and the extracellular extracts (EC) were filtered through syringe filter of 0.20 μm pore size housing with PTFE membrane (Millipore). The total polyketide secondary metabolite content extracted from the mycelial biomass was analysed by measuring the absorbance of all the extracts by spectral analysis using a UV–visible spectrophotometer (UV-1800, Shimadzu Corporation, Tokyo, Japan) at 276 nm (i.e., the λ_max_ of the target naphtoquinone pigments bikaverin and norbikaverin), and expressed in terms of milli-equivalents (meqv.) of polyketide metabolites per gram of dry cell mass (meqv g^−1^), or in terms of meqv. per liter of culture medium (i.e., volumetric production in meqv L^−1^ in the culture medium), according the method described by Lebeau et al. (2017). In a similar manner, the volumetric production of polyketide extrolites secreted by the strain in the culture filtrate (supernatant) was estimated by a spectrophotometer on the basis of the measured absorbance at 276 nm, and expressed as meqv. of polyketide extrolites per liter of culture filtrate (Lebeau et al. 2017). This estimation is a value proportional to the total polyketide concentration in the culture filtrate. Indeed, most polyketide-derived molecules (such as naphthoquinone pigments and other *Fusarium* mycotoxins) are characterized by absorption bands in the UV domain (near 240–260 nm) due to the benzene structure and most of them presented one UV absorption maxima near 280 nm (examples include the pigments bikaverin and norbikaverin, which exhibited a λ_max_ of t_max_ at 276 nm), whereas absorbance in the visible region (390–710 nm) highly depends on the nature and number of the substituted groups. All experiments were conducted in triplicate. The extracts were then stored at 4 °C in an amber vial prior to chromatographic analysis.

### High-performance liquid-chromatography combined with photodiode array-detection (HPLC–DAD) analyses

Reverse phase HPLC–DAD analysis was performed on each IC and EC extract (25 μL injection) using a Dionex HPLC–DAD system (Ultimate 3000 apparatus, Dionex, Germering, Germany). The separation was performed on a 2.1 mm i.d. × 150 mm, 5 μm Hypersil Gold™ column (Thermo Scientific Inc., Waltham, MA, USA) at 30 °C with a water-acetonitrile-formic acid gradient system, according to the analytical method described by Lebeau et al. (2017). Monitoring, data recording, and processing were carried out with the Chromeleon v.6.80 software (Dionex). Solvents (acetonitrile, methanol and ethanol, 99.9%-HPLC quality) and formic acid (purity 99%) were obtained from Carlo Erba (Val de Reuil, France). Bikaverin standard from *Fusarium subglutinans* (1 mg, purity ≥ 98%) for HPLC–DAD analyses was obtained from Sigma-Aldrich (Merck KGaA).

### UHPLC-high-resolution mass spectrometry (HRMS) analyses

The secondary metabolites isolated in IC and EC extracts of *F. oxysporum* were identified by UHPLC-HRMS. Analyses were performed on an Agilent 1290 Infinity LC system with a DAD detector, coupled to an Agilent 6550 iFunnel Q-TOF with an electrospray ionization source (Agilent Technologies, Santa Clara, CA, USA). The separation was performed on a 2.1 mm i.d. × 250 mm, 2.7 μm Poroshell 120 Phenyl-Hexyl column (Agilent) at 60 °C with a water-acetonitrile gradient (both buffered with 20 mM formic acid), according to the method described by Klitgaard et al. (2014).

## Results

**Fig. 1:**
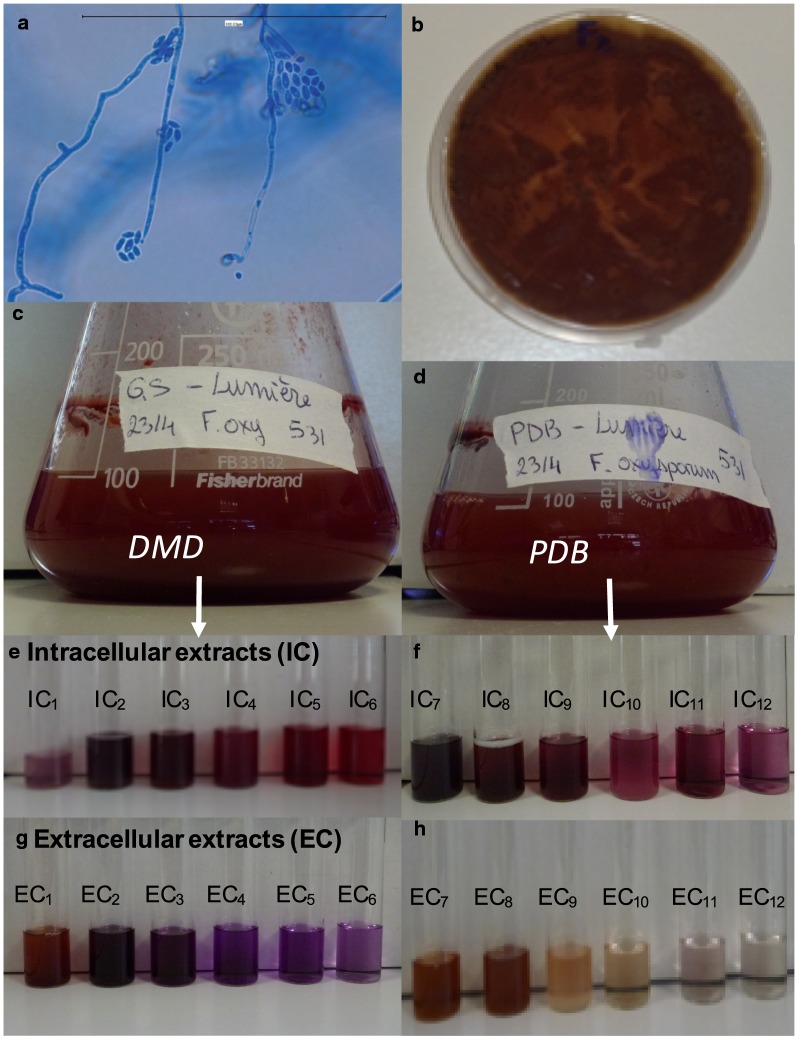
**a** Morphology of the filamentous fungus *Fusarium oxysporum*, **b** grown on potato-dextrose-agar (PDA) plate, **c** in submerged culture of defined minimal dextrose broth (DMD), and **d** in potato-dextrose broth (PDB). Intracellular (IC) and extracellular (EC) liquid extracts obtained using a 6-stage pressurized liquid extraction method with various polarity profile on lyophilized mycelial cells (**e**, **f**) and lyophilized culture filtrate (**g**, **h**), respectively

The strain *F. oxysporum* LCP531 (Fig. 1a, b) was cultivated 7 days in PDB (Fig. 1c) and DMD (Fig. 1d) submerged cultures. The volumetric production of biomass (in g L^−1^) and polyketide extrolites (in meqv L^−1^ of culture broth), and the intracellular production of polyketide compounds (in meqv g^−1^ of dry biomass, and in meqv L^−1^ of culture broth) from *Fusarium oxysporum* LCP 531 strain grown in PDB and DMD medium for 7 days, under light exposure or darkness, are presented in Table 1. The fungal metabolites from the mycelial biomass and from the lyophilized culture filtrate were extracted and fractionated by pressurized liquid extraction at higher pressure using a 6-stage decreasing solvent polarity profile (starting from an extraction solvent polarity index from 10.0 to 4.0) as described in “Materials and methods” section. Such polarity sequence allowed to perform a refined isolation of the different fungal secondary metabolites, based on their specific polarity profiles. Therefore, and as the fungal biomass was likely to contain a mixture of secondary metabolites of various natures, a multistage extraction method using solvents of different polarity was likely to be required, to obtain the most exhaustive secondary metabolites composition profile (Caro et al. 2017; Lebeau et al. 2017). A series of six intracellular extracts (IC; Fig. 1e, f) and six extracellular extracts (EC; Fig. 1g, h) were collected, and their composition of fungal secondary metabolites were first characterized by HPLC–DAD and then, all the metabolites were identified by UHPLC-HRMS.

**Table 1:** Volumetric production of biomass (in g L^−1^), polyketide extrolites (in meqv L^−1^) and intracellular production of polyketide compounds (in meqv g^−1^ of dry biomass, or in meqv L^−1^ of culture broth) from the *Fusarium oxysporum* LCP531 strain grown in PDB and DMD medium for 7 days, under light exposure or darkness

Broth	Exposition	Biomass (in g L^−1^)	Polyketide extrolites (in meqv. L^−1^)	Intracellular production of polyketide compounds	
				(meqv. g^−1^ biomass)	(in meqv. L^−1^ culture broth)
DMD	Light	4.63 ± 0.25	86 ± 5.2	36.2 ± 1.4	166 ± 9.1
DMD	Darkness	3.73 ± 0.21	70 ± 4.8	34.3 ± 1.6	127 ± 6.3
PDB	Light	3.70 ± 0.15	135 ± 9.2	35.9 ± 1.8	133 ± 7.9
PDB	Darkness	3.43 ± 0.20	166 ± 11.5	26.8 ± 1.5	91 ± 4.6

### Identification of the bikaverin and intermediates thereof in *Fusarium oxysporum* LCP531

**Fig. 2:**
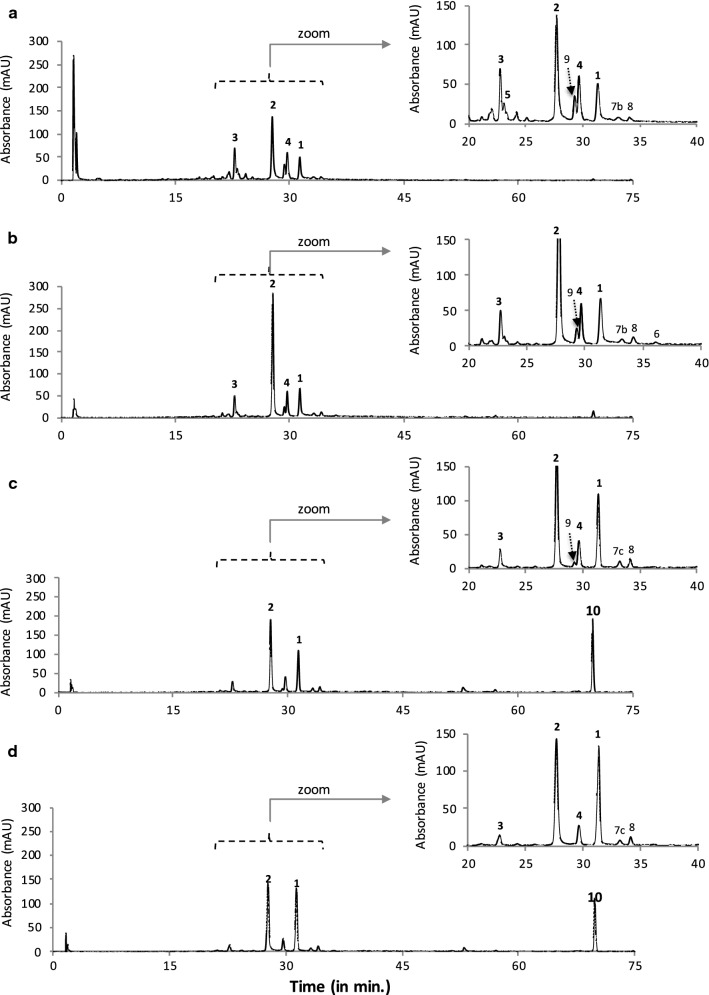
HPLC–DAD chromatograms of intracellular extracts (IC) of *F. oxysporum* LCP531 mycelial cells grown on define minimal dextrose broth (DMD) and extracted with **a** 50% aqueous methanol (extract IC_2_ shown in Fig. 1e), **b** then 50% aqueous ethanol (extract IC_3_), **c** > 99.9% methanol (extract IC_4_), and **d** MeOH:EtOH, 1:1, v/v (extract IC_5_) as extraction solvents. The IC samples described here were considered as the most representative due to the pigment composition and intensity of their coloration as shown in Fig. 1. Assignment of the bikaverin **1** and possible intermediates **2**–**6** (norbikaverin **2**, oxo-pre-bikaverin **3**, me-oxo-pre-bikaverin **4**, dinor-bikaverin **5**, pre-bikaverin **6**) and the ergosterol-derivate **10** were done by HRMS according to their mass to charge ratio. The minor secondary metabolites, labelled as compounds **7a**, **7b**, **7c**, **8** and/or **9** in the chromatograms were isolated but not identified

**Fig. 3:**
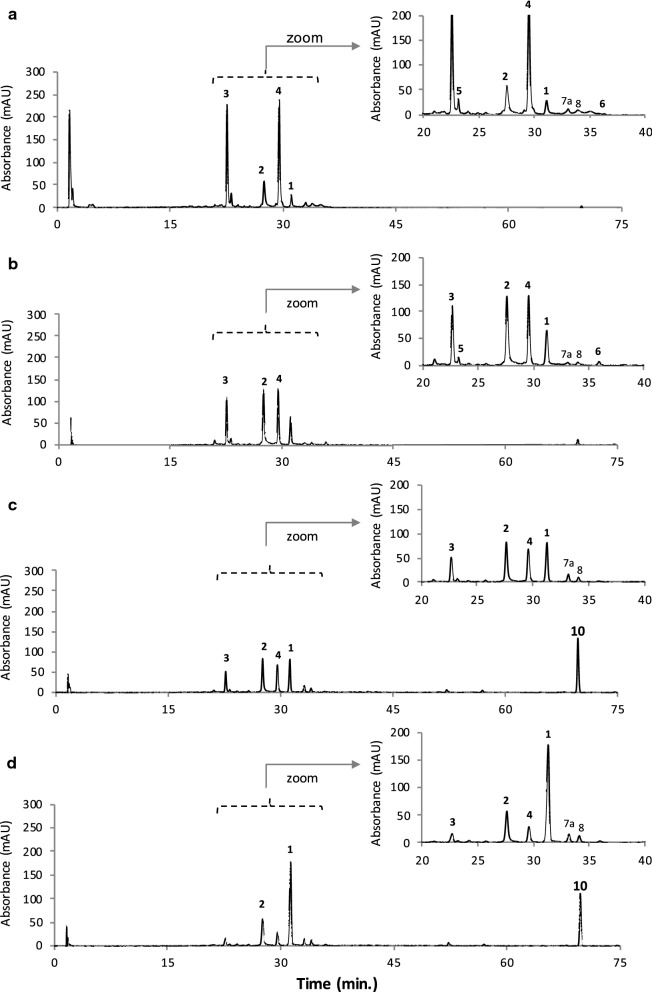
HPLC-DAD chromatograms of intracellular extracts (IC) of *F. oxysporum* LCP531 mycelial cells grown on potato dextrose broth (PDB) and extracted with **a** 50% aqueous methanol (extract IC_8_ shown in Fig. 1f), **b** then 50% aqueous ethanol (extract IC_9_), **c** > 99.9% methanol (extract IC_10_), and **d** MeOH:EtOH, 1:1, v/v (extract IC_11_) as extraction solvents. See Fig. 2 for the legends

The HPLC–DAD chromatograms of the four more deeply colored intracellular extracts of *F.* *oxysporum* LCP531 from mycelium grown in either DMD (IC_2-5_) or PDB broths (IC_8-11_) are shown in Figs. 2 and 3, respectively. The more intensively pigmented fractions were obtained when extracted with solvent mixtures displaying medium–high polarity solvent profile such as 50% aqueous methanol, v/v (Figs. 2a, 3a) and 50% aqueous ethanol, v/v (Figs. 2b, 3b), or with medium–low polarity solvent profile such as 100% methanol (Figs. 2c, 3c) and MeOH:EtOH, 1:1; v/v (Fig. 2d, 3d) (e.g., corresponding to the extracts numbered IC_2_ to IC_5_, and IC_8_ to IC_11_ shown in Fig. 1e, f), which was not surprising regarding the chemical structures of reported polyketidic pigments. This suggests that these extracts are likely to contain a larger panel of pigmented compounds produced by *F. oxysporum* LCP531, and were thus further analysed.

**Fig. 4:**
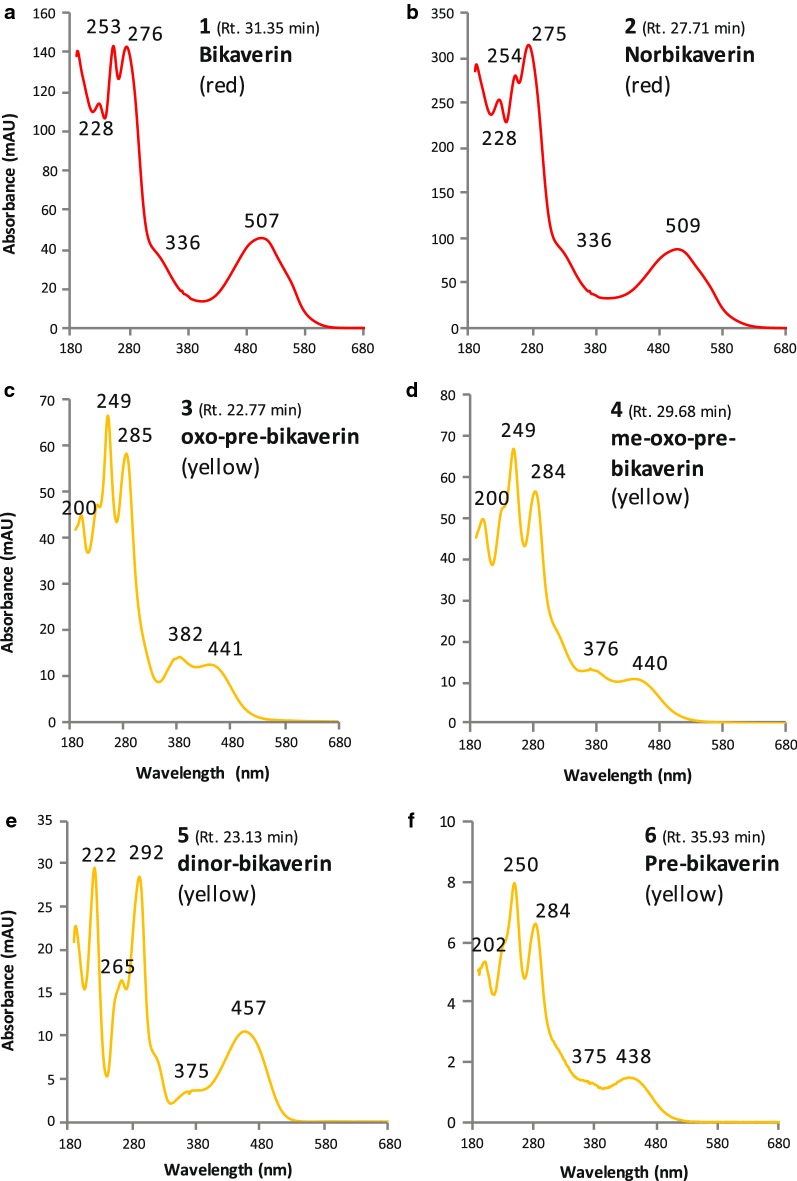
Absorbance spectra of the naphthoquinone pigments **a** bikaverin **1** and bikaverin intermediates: **b** norbikaverin **2**, **c** oxo-pre-bikaverin **3**, **d** me-oxo-pre-bikaverin **4**, **e** dinor-bikaverin **5**, and **f** pre-bikaverin **6**, isolated in intracellular extracts of *F. oxysporum* LCP531. Numbers and retention time (Rt.; in min.) are referring to the compounds identified on Figs. 2 and 3. Colors on graphs are referring to the red or yellow pigments isolated and identified in this study

First, and as mentioned in our previous study (Lebeau et al. 2019), the results (Table 2) confirmed that bikaverin **1** (6,11-dihydroxy-3,8-dimethoxy-1-methylbenzo[b]xanthene-7,10,12 -trione) and norbikaverin **2** (6,10,11-trihydroxy-3-methoxy-1-methylbenzo [b]xanthene-7,8,12-trione) were the major red naphthoquinone pigments (with λ_max_ at 276 nm in the UV region, and an absorption maxima in the visible region of the spectrum at ca. 510 nm) that could be isolated from mycelial extracts obtained from either DMD or PDB broth (Table 2). We also demonstrated that they were responsible for the intense red shades previously reported in IC extracts as shown in Fig. 1e, f. The chemical structure of bikaverin **1** and norbikaverin **2** (both naphthoquinone pigments with nonaketide naphthazarin quinone structure) was identified here by high-resolution mass spectrometry (HRMS) performed on the different IC extracts of the *F.* *oxysporum* LCP531 strain. Bikaverin **1,** which exhibited λ_max_ at 195, 228, 253, 276, 336, and 507 nm (Fig. 4a), showed a *m/z* value of 383.0292 in positive mode [M+H]^+^ (Additional file 1: Fig. S1), while norbikaverin 2 exhibiting λ_max_ at 196, 228, 254, 275, 336, and 509 nm (Fig. 4b), showed a ESI–MS molecular ion at *m/z* 369.0147 [M+H]^+^ (Additional file 1: Fig. S2) in good agreement with the expected mass of the standards (Nielsen and Smedsgaard 2003; Wiemann et al. 2009; Arndt et al. 2015).

**Table 2:** Intracellular (IC) secondary metabolites including naphthoquinone pigments (expressed in volumetric production in meqv L^−1^ of culture broth) extracted by pressured liquid extraction from the mycelia of the *Fusarium oxysporum* LCP531 strain grown either in DMD or PDB broth, according to the polarity index of the extraction solvent

Compound no	Colour	λ_max_ in visible (in nm)	*m/z* [M+H]^+^ (in g mol^−1^)	Extraction solvent (amount of the metabolites in each extract)						Total amount extracted (in meqv L^−1^ of culture broth)
				H_2_O	50% MeOH	50% EtOH	MeOH	MeOH/EtOH	EtOH	
Solvent polarity index:				10.0	7.5	7.0	5.0	4.5	4.0	
Mycelium grown in DMD										
Bikaverin **1**	Red	507	383	0.5 ± 0.1	3.7 ± 0.2	4.8 ± 0.4	7.1 ± 0.4	10.3 ± 0.8^a^	4.8 ± 0.3	31.2 ± 2.2
Norbikaverin **2**	Red	509	369	< 0.1	11.2 ± 0.8	20.4 ± 1.2^b^	13.0 ± 0.9	12.1 ± 0.6	4.8 ± 0.2	61.5 ± 3.7
Oxo-pre-bikaverin **3**	Yellow	382, 441	339	< 0.1	2.7 ± 0.2^c^	2.1 ± 0.2	1.6 ± 0.2	1.1 ± 0.1	0.2 ± 0.1	7.7 ± 0.8
Me-oxo-pre-bikaverin **4**	Yellow	376, 440	353	< 0.1	3.8 ± 0.3^d^	3.6 ± 0.2	2.7 ± 0.3	1.7 ± 0.2	0.9 ± 0.2	12.6 ± 1.2
Dinor-bikaverin **5**	Yellow	457	357	< 0.1	0.7 ± 0.2	< 0.1	< 0.1	< 0.1	< 0.1	0.7 ± 0.2
Pre-bikaverin **6**	Yellow	438	323	< 0.1	< 0.1	0.2 ± 0.1	< 0.1	< 0.1	< 0.1	0.2 ± 0.1
Pigment ni **7a**	Yellow	376, 437	nd	nd	nd	nd	nd	nd	nd	nd
Pigment ni **7b**	Purple	496, 523	nd	< 0.1	0.4 ± 0.1	0.5 ± 0.1	< 0.1	< 0.1	< 0.1	1.0 ± 0.2
Pigment ni **7c**	Purple	491, 530	nd	< 0.1	< 0.1	< 0.1	0.7 ± 0.2	0.5 ± 0.1	0.3 ± 0.1	1.5 ± 0.4
Pigment ni **8**	Yellow	430	nd	< 0.1	0.4 ± 0.1	0.7 ± 0.2	0.8 ± 0.1	0.7 ± 0.1	0.7 ± 0.2	3.3 ± 0.7
Pigment ni **9**	Purple	516, 549	nd	< 0.1	1.9 ± 0.2	1.3 ± 0.3	0.2 ± 0.1	< 0.1	< 0.1	3.4 ± 0.5
Total pigments extracted (in meqv L^−1^ culture broth)				0.5 ± 0.3	24.8^e^ ± 2.1	33.6^f^ ± 2.7	26.2^g^ ± 2.2	26.4^h^ ± 1.9	11.6 ± 1.1	123.1 ± 10.3
> Other metabolites extracted										
Beauvericin		–	784	nd	nd	nd	nd	nd	nd	nd
Ergosterol derivate		–	393	< 0.1	< 0.1	0.9 ± 0.1	12.8 ± 1.2	7.3 ± 0.4	0.5 ± 0.1	21.6 ± 1.8
Others (ni)		–	nd	5.8 ± 0.4	12.8 ± 0.8	0.3 ± 0.1	0.5 ± 0.1	0.6 ± 0.2	1.3 ± 0.3	21.3 ± 1.9
Mycelium grown in PDB										
Bikaverin **1**	Red	507	383	1.0 ± 0.3	1.2 ± 0.2	2.3 ± 0.2	2.8 ± 0.5	7.8 ± 0.7^a^	2.3 ± 0.3	17.6 ± 2.1
Norbikaverin **2**	Red	509	369	0.5 ± 0.1	2.3 ± 0.3	5.6 ± 0.4^b^	3.4 ± 0.3	2.7 ± 0.2	0.6 ± 0.1	15.1 ± 1.4
Oxo-pre-bikaverin **3**	Yellow	382, 441	339	< 0.1	7.1 ± 0.4^c^	3.6 ± 0.3	1.7 ± 0.4	0.6 ± 0.2	< 0.1	13.0 ± 1.3
Me-oxo-pre-bikaverin **4**	Yellow	376, 440	353	< 0.1	8.3 ± 0.4^d^	3.7 ± 0.2	2.2 ± 0.2	1.0 ± 0.3	< 0.1	15.3 ± 1.1
Dinor-bikaverin **5**	Yellow	457	357	< 0.1	0.6 ± 0.1	0.3 ± 0.1	0.1 ± 0.1	< 0.1	< 0.1	1.1 ± 0.3
Pre-bikaverin **6**	Yellow	438	323	< 0.1	< 0.1	0.2 ± 0.2	< 0.1	< 0.1	< 0.1	0.3 ± 0.2
Pigment ni **7a**	Yellow	376, 437	nd	< 0.1	0.3 ± 0.1	0.2 ± 0.1	0.5 ± 0.2	0.5 ± 0.2	< 0.1	1.5 ± 0.6
Pigment ni **7b**	Purple	496, 523	nd	nd	nd	nd	nd	nd	nd	nd
Pigment ni **7c**	Purple	491, 530	nd	nd	nd	nd	nd	nd	nd	nd
Pigment ni **8**	Yellow	430	nd	< 0.1	0.2 ± 0.1	0.2 ± 0.1	0.3 ± 0.1	0.4 ± 0.1	< 0.1	1.1 ± 0.4
Pigment ni **9**	Purple	516, 549	nd	nd	nd	nd	nd	nd	nd	nd
Total pigments extracted (in meqv L^−1^ culture broth)				1.5 ± 0.3	20.1^i^ ± 1.6	16.0^j^ ± 1.6	11.2^k^ ± 1.8	13.2^l^ ± 1.7	3.0 ± 0.4	65.0 ± 7.4
> Other metabolites extracted										
Beauvericin		–	784	nd	nd	nd	nd	nd	nd	nd
Ergosterol derivate		–	393	< 0.1	< 0.1	0.4 ± 0.2	5.0 ± 0.4	4.1 ± 0.3	0.7 ± 0.2	10.3 ± 1.1
Others (ni)		–	nd	48.2 ± 2.9	5.6 ± 0.3	2.1 ± 0.3	0.3 ± 0.2	0.4 ± 0.2	0.9 ± 0.3	57.5 ± 4.2

Underlined data highlighted the best extraction solvent for each considered secondary metabolite

*ni* not identified, *nd* not determined

^a^Bikaverin is preferentially extracted by solvent with polarity index (p.i.) of 4.5; ^b^ Norbikaverin by solvent p.i. of 7.0; ^c,d^ Oxo-pre-bikaverin and Me-oxo-pre-bikaverin by solvent p.i. of 7.5; ^e^ corresponding to the extract IC_2_ shown in Fig. 1e and HPLC-DAD chromatogram shown in Fig. 2a; ^f^ extract IC_3_ in Fig. 1e and chromatogram in Fig. 2b; ^g^ extract IC_4_ in Fig. 1e and chromatogram in Fig. 2c; ^h^ extract IC_5_ in Fig. 1e and chromatogram in Fig. 2d; ^i^ extract IC_8_ shown in Fig. 1e and chromatogram in Fig. 3a; ^j^ extract IC_9_ in Fig. 1e and chromatogram in Fig. 3b; ^k^ extract IC_10_ in Fig. 1e and chromatogram in Fig. 3c; ^l^ extract IC_11_ shown in Fig. 1e and chromatogram in Fig. 3d

Interestingly, two others major naphthoquinone pigments, compounds labelled as **3** and **4** in chromatograms (Figs. 2 and 3), with absorption maxima at ca. 440 nm in visible-light domain (suggested yellow pigments) were detected in mycelial extracts of the *F.* *oxysporum* LCP531 (Table 2). The UV–visible spectra of the four major naphthoquinone pigments, e.g. bikaverin **1**, norbikaverin **2,** and compounds **3** and **4**, isolated from *F.* *oxysporum* LCP531 mycelial cells cultured either on DMD or PDB were detailed in Fig. 4a–d. These two compounds **3** and **4** were mostly extracted from mycelial biomass with 50% aqueous methanol as the extracting solvent, as shown in Table 2  (underlined data), suggesting higher polarity profiles compared to bikaverin **1** which was preferentially extracted with medium–low polarity solvent (MeOH:EtOH, 1:1; v/v; underlined data in the Table 2).

The results indicated a clear impact of the nutrients profile on the *F. oxysporum* LCP531 pigmentation, when comparing pigment yields on PDB and DMD (Table 2). Indeed, twice as much bikaverin **1** (31.2 meqv L^−1^, about 25% w/w of the total pigments extracted in DMD) and norbikaverin **2** (61.5 meqv L^−1^, about 50% w/w of total pigment extracted) was obtained from the mycelial cells when *F.* *oxysporum* LCP531 was grown on DMD broth culture as compared to intracellular concentrations obtained on PDB: that is only 17.6 and 15.1 meqv L^−1^ of bikaverin **1** and norbikaverin **2**, respectively. Surprisingly, norbikaverin **2**, which is usually occurring as a minor compound as being the precursor of the pigment bikaverin **1** in the polyketide biosynthesis pathway described in *Fusarium* species, was observed in this study as the major pigmented intracellular molecule (50% w/w total pigment extracted) produced by *F.* *oxysporum* LCP531 grown in DMD broth (i.e., with simple carbon and nitrogen sources readily available). Indeed, the amount of norbikaverin **2** detected was not as negligible as one might have expected. About 37% w/w of norbikaverin **2** over the total secondary metabolites extracted (pigments and others) from dry cell weight grown in DMD broth was detected, for only 18% w/w of bikaverin **1** (Fig. 2, Table 2). In contrast, concentrations of the pigments **3** and **4** were higher in mycelial biomass cultured in PDB broth (Fig. 3, Table 2) than in DMD broth (Fig. 2, Table 2). Their intracellular concentrations in PDB broth culture were estimated at 13.0 and 15.3 mg L^−1^, respectively, for the pigments **3** and **4.**

Until today, the characterization of these pigments **3** and **4** were never identified from culture of *F.* *oxysporum*; and unfortunately in our previous study, we were not able to identify their chemical structure (Lebeau et al. 2019). Here, the analysis of the HRMS results led us to the assumption that the compound **3** was likely to be the naphthoquinone oxo-pre-bikaverin **3**, which was newly characterized by Arndt and co-workers (2015) as a red bikaverin intermediate produced by *F. fujikuroi*. Indeed, the absorption spectrum (λ_max_ 200, 249, 285, 382, 441 nm; Fig. 4c) and to the ESI–MS molecular ion observed at *m/z* 339.0070 in positive mode [M+H]^+^ (Additional file 1: Fig. S3) obtained while analysed the compound **3**, were matching the expected mass of the oxo-pre-bikaverin previously analysed by the authors (Arndt et al. 2015). The compound **4,** which exhibited λ_max_ at 200, 249, 284, 376, and 440 nm (Fig. 4d), and showed a *m/z* value of 353.0205 in positive mode [M+H]^+^ (Additional file 1: Fig. S4) was assumed to be another bikaverin intermediate, me-oxo-pre-bikaverin **4**, according to the HRMS data published by Arndt and co-workers (2015) for the similar molecule detected in fungal extracts of *F. fujikuroi*. To the best of our knowledge, this is the first report of the simultaneous isolation and identification of oxo-pre-bikaverin **3** and me-oxo-pre-bikaverin **4** in submerged culture of *F.* *oxysporum*.

Furthermore, another two minor naphthoquinone pigments, e.g., the compounds **5** and **6** as shown in chromatograms (Figs. 2a, b and 3a, b) were isolated and structurally identified in the IC extracts of *F.* *oxysporum* (Table 2). The compound **5** is assumed to be the molecule dinor-bikaverin **5** (6-hydroxy-7,10-diketo-pre -bikaverin) according to both its absorption spectrum, which exhibited λ_max_ at 193, 222, 265, 292, 375, and 457 nm (Fig. 4e) and the ESI–MS molecular ion observed at *m/z* 357.0520 in positive mode [M+H]^+^ (Additional file 1: Fig. S5), which is relatively close to those of the dinor-bikaverin described by Arndt and co-workers (2015) reporting a *m/z* value of 355.0448 for the exact mass of dinor-bikaverin isolated from *F.* *fujikuroi*. The compound **6** is assumed to be the molecule pre-bikaverin **6** according to its absorption spectrum which exhibited λ_max_ at 202, 250, 284, 375, and 438 nm (Fig. 4f) and to the ESI–MS molecular ion observed at *m/z* 323.1049 in positive mode [M+H]^+^ (Additional file 1: Fig. S6) relatively close to those of the pre-bikaverin detected from *F.* *fujikuroi* and described first by Ma et al. (2007) and then by Arndt and co-workers (2015). Additionally, this is also the first report of the concomitant identification of these two bikaverin intermediates, e.g. dinor-bikaverin **5** and pre-bikaverin **6**, in culture of the current *F.* *oxysporum* LCP531.

As shown in the chromatograms of the IC extracts of *F.* *oxysporum* LCP531 (Figs. 2 and 3), other minor secondary metabolites, labelled as compounds **7a**, **7b**, **7c, 8** and **9** (Additional file 1: Figs. S7–S11), were isolated but not identified, and further experiments with dereplication purpose using UHPLC-HRMS were unsuccessful for their identifications (no HRMS spectra in ESI  positive or negative mode, signal is too weak). Surprisingly, three of these minor uncharacterized compounds, e.g., the compounds **7b**, **7c,** and **9** (exhibiting absorption maxima from 510 to 550 nm, i.e., corresponding to a purple hue), were exclusively detected in mycelial extracts of *F.* *oxysporum* LCP531 cultured in DMD broth (Fig. 2; Table 2), as recently reported in our previous study (Lebeau et al. 2019).

**Fig. 5:**
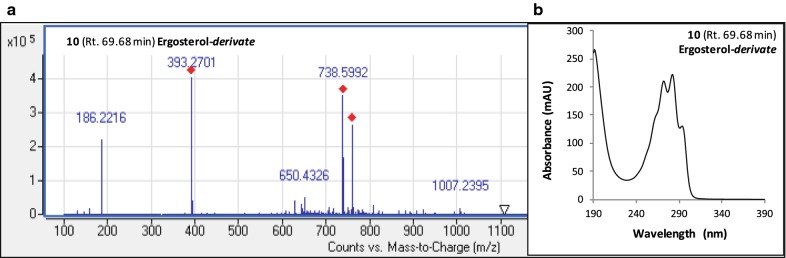
Mass (**a**) and absorbance (**b**) spectra of the ergosterol-derivate isolated in *F.* *oxysporum* LCP531 mycelial cells. Number and retention time (Rt.; in min.) are referring to compound identified on Figs. 2 and 3

Finally, an ergosterol-derivate **10** was also isolated in chromatograms of the IC extracts of the fungus cultured either in DMD or PDB broth (Figs. 2 and 3, respectively). In fact, the absorbance and HRMS spectra of this molecule (shown in Fig. 5) showed similar profile to the known compound ergosterol (Additional file 1: Fig. S12a), with an ESI–MS molecular ion observed at *m/z* 393 in positive mode [M+H]^+^ which was relatively close to those of the standard molecule reported elsewhere; the molecule, which in addition to the expected molecular ion at *m/z* 397 [M+H]^+^, also yielded the same ion at *m/z* 393 (major) because ergosterol had undergone desaturation during LC–MS according to Slominski et al. (2005) and Dame et al. (2016). This fungal secondary metabolite was best extracted with medium–low polarity profile solvent mixture such as pure methanol (Table 2). The intracellular concentration of this ergosterol-derivate was estimated at 21.6 meqv L^−1^ of culture broth (13% w/w of total secondary metabolites on DMD) and 10.3 meqv L^−1^ (7.8% w/w of total secondary metabolites on PDB) for fungal submerged culture in DMD and PDB broth, respectively.

### *Characterization of the major extrolites produced by F. oxysporum* LCP531

HPLC–DAD chromatographic analyses carried out on the extracellular (EC) extracts obtained from the lyophilized fermentation broths (culture supernatants) for *F.* *oxysporum* LCP531 culture in DMD (Additional file 1: Fig. S13-A) also demonstrated that the pigment bikaverin **1** was the major secondary metabolite excreted and isolated in the purple-colored extracellular extracts of *F. oxysporum* LCP531 (e.g., the extracts labelled EC_2_ to EC_6_ shown in Fig. 1e). Extracellular production of bikaverin **1** yielded at 55.1 meqv L^−1^ of culture broth (65% w/w of the total diffusible extrolites isolated in the 7-day-old DMD fermentation broths) (Table 3). By contrast, chromatographic analyses performed on the EC extracts from the lyophilized 7-day-old PDB fermentation broths of *F.* *oxysporum* LCP531 (Additional file 1: Fig. S13-B) indicated that the well-known toxin beauvericin (BEA) (Additional file 1: Fig. S12b) (Logrieco et al. 1998; Zhan et al. 2007) was the major extrolite isolated; Its extracellular production was estimated at 97.1 meqv L^−1^ of culture broth (Table 3). BEA was characterized by its absorption λ_max_ at 210 nm and an ion at *m/z* 784 in positive mode [M+H]^+^ (together with characteristic ion at m/z 806 [M+Na]^+^) in good agreement with the expected mass of the standard. This indicated that active secretion of diffusible bikaverin and intermediates was occurring under the DMD nutrient condition in shake flask cultures (i.e. glucose and ammonium sulfate, combined with sufficient concentrations of salts and bio-elements), without major coproduction of *Fusarium* mycotoxins like beauvericin. By contrast, in liquid medium like PDB containing complex nitrogen (amino acids and proteins from potato) and carbon (starch) sources, our results indicated that the *F.* *oxysporum* LCP531 strain favored the synthesis of other bikaverin intermediates in mycelial biomass, mainly the two naphthoquinones, oxo-pre-bikaverin **3** and me-oxo-pre-bikaverin **4** (Additional file 1: Fig. S13-B; Table 3). Additionally, the liberation of BEA as the major extrolite in the PDB fermentation broth was also reported.

**Table 3:** Extracellular (EC) secondary metabolites including naphthoquinone pigments (expressed in volumetric production in meqv L^−1^ of culture broth) extracted by pressured liquid extraction from the 7-day-old fermentation broth (culture supernatant) of the *Fusarium oxysporum* LCP531 strain grown either in DMD or PDB broth, according to the polarity index of the extraction solvent

Compound no	Colour	λ_max_ in visible (in nm)	m/z [M+H]^+^ (in g mol^−1^)	Extraction solvent (amount of the metabolites in each extract)						Total amount extracted from mycelium (in meqv L^−1^ of culture broth)
				H_2_O	50% MeOH	50% EtOH	MeOH	MeOH/EtOH	EtOH	
Solvent polarity index:				10.0	7.5	7.0	5.0	4.5	4.0	
Culture supernatant from DMD										
Bikaverin **1**	Red	507	383	< 0.1	34.6 ± 2.1	9.6 ± 0.8	5.6 ± 0.7	3.8 ± 0.4	1.4 ± 0.4	55.1 ± 4.4
Norbikaverin **2**	Red	509	369	< 0.1	6.5 ± 0.4	3.5 ± 0.3	1.1 ± 0.3	0.8 ± 0.2	0.4 ± 0.2	12.3 ± 1.4
Oxo-pre-bikaverin **3**	Yellow	382, 441	339	< 0.1	1.0 ± 0.2	0.6 ± 0.2	0.3 ± 0.1	0.1 ± 0.1	0.1 ± 0.1	2.1 ± 0.7
Me-oxo-pre-bikaverin **4**	Yellow	376, 440	353	< 0.1	1.1 ± 0.2	0.7 ± 0.1	0.3 ± 0.2	0.2 ± 0.1	0.1 ± 0.1	2.4 ± 0.7
Dinor-bikaverin **5**	Yellow	457	357	< 0.1	0.2 ± 0.1	0.1 ± 0.1	0.1 ± 0.1	< 0.1	< 0.1	0.3 ± 0.3
Pre-bikaverin **6**	Yellow	438	323	< 0.1	< 0.1	0.2 ± 0.1	< 0.1	< 0.1	< 0.1	0.2 ± 0.1
Pigment ni **7b**	Purple	496, 523	nd	< 0.1	< 0.1	< 0.1	0.1 ± 0.1	0.1 ± 0.1	< 0.1	0.1 ± 0.1
Pigment ni **8**	Yellow	430	nd	< 0.1	< 0.1	0.4 ± 0.1	0.1 ± 0.1	0.4 ± 0.1	0.4 ± 0.1	1.3 ± 0.4
Total pigments extracted (in meqv L^−1^ culture broth)				< 0.1	43.4 ± 3.0	15.1 ± 1.7	7.4 ± 1.6	5.4 ± 1.0	2.5 ± 0.9	73.7 ± 8.2
> Other extrolites extracted										
Beauvericin		–	784	nd	nd	nd	nd	nd	nd	nd
Ergosterol derivate		–	393	nd	nd	nd	nd	nd	nd	nd
Others (ni)		–	nd	12.3 ± 1.0	< 0.1	< 0.1	< 0.1	< 0.1	< 0.1	12.3 ± 1.0
Culture supernatant from PDB										
Bikaverin **1**	Red	507	383	< 0.1	< 0.1	< 0.1	0.7 ± 0.3	0.3 ± 0.1	0.5 ± 0.1	1.5 ± 0.5
Norbikaverin **2**	Red	509	369	< 0.1	< 0.1	< 0.1	0.1 ± 0.1	0.1 ± 0.1	0.1 ± 0.1	0.3 ± 0.3
Oxo-pre-bikaverin **3**	Yellow	382, 441	339	0.8 ± 0.2	8.9 ± 0.8	2.9 ± 0.3	0.3 ± 0.1	0.3 ± 0.2	0.1 ± 0.1	13.3 ± 1.7
Me-oxo-pre-bikaverin **4**	Yellow	376, 440	353	1.3 ± 0.4	8.7 ± 0.6	3.4 ± 0.4	1.2 ± 0.4	0.5 ± 0.2	0.3 ± 0.1	15.3 ± 2.1
Dinor-bikaverin **5**	Yellow	457	357	1.1 ± 0.2	3.1 ± 0.4	1.1 ± 0.4	0.1 ± 0.1	0.1 ± 0.1	< 0.1	5.5 ± 1.2
Pre-bikaverin **6**	Yellow	438	323	< 0.1	< 0.1	< 0.1	< 0.1	< 0.1	< 0.1	< 0.1
Pigment ni **7a**	Yellow	376, 437	nd	< 0.1	< 0.1	0.3 ± 0.1	0.2 ± 0.1	< 0.1	< 0.1	0.6 ± 0.2
Pigment ni **8**	Yellow	430	nd	< 0.1	< 0.1	0.7 ± 0.2	0.4 ± 0.1	0.1 ± 0.1	0.1 ± 0.1	1.3 ± 0.5
Total pigments extracted (in meqv L^−1^ culture broth)				3.1 ± 0.8	20.7 ± 1.8	8.4 ± 1.4	3.1 ± 1.2	1.5 ± 0.8	1.2 ± 0.5	37.9 ± 6.5
> Other extrolites extracted										
Beauvericin		–	784	39.1 ± 2.1	47.2 ± 2.5	10.7 ± 0.6	< 0.1	< 0.1	< 0.1	97.1 ± 5.2
Ergosterol derivate		–	393	nd	nd	nd	nd	nd	nd	nd
Others (ni)		–	nd	nd	nd	nd	nd	nd	nd	nd

## Discussion

*Fusarium* strains are well known to be one of the most widely diverse and dispersed fungal strains. Bikaverin production has been described as a common trait amongst *Fusarium* species (review in: Limón et al. 2010; Lale and Gadre 2016; Lebeau et al. 2019), while more rarely occurring in other fungi. Bikaverin is classified as a mycotoxin, even if its occurrence was observed in non-virulent *Fusarium* sp. Although the *Fusarium* species, such as *F.* *oxysporum,* that produce bikaverin are commonly considered as phytopathogens with great economic and agricultural importance, the presence of the pigment has not been found to be related to the phytopathogenic activity. The effects of bikaverin are versatile upon the organisms (Limón et al. 2010). Despite its classification as a contaminant in food and feed, there are no reports to this date of harmful effects of products containing bikaverin on human or animal health, although appropriate toxicological studies will still be required in order to ensure its complete safe use in any future applications (Norred et al. 1992). Interestingly though, bikaverin was proven to have antibiotic effects against diverse organisms, particularly on protozoa (*Leishmania brasiliensis*; Balan et al. 1970), on pine wood nematode (*Bursaphelenchus xylophilus;* Kwon et al. 2007), on tomato late blight caused by *Phytophthora infestans* (Kim et al. 2007) and on some filamentous fungal strains. However, it was concluded to have higher toxicity activity once combined with fusaric acid or other mycotoxins (Kwon et al. 2007), suggesting that bikaverins could serve as potent biocontrol agent for agricultural uses.

Additionally, recent studies highlighted the putative anti-cancer and anti-tumor properties of bikaverin-like compounds (Haidar et al. 2017, 2019), suggesting potentialities for drugs development and pharmaceutical applications in human health, and consequently strengthening the need to confirm the consistency of bikaverin biosynthesis pathway and its intermediates in various *Fusarium* strains. Bikaverin has been reported in various *Fusarium* species (Chelkowski et al. 1992), but the only reported biosynthetic route for bikaverin has been extensively and exclusively elucidated in *Fusarium fujikuroi* (Arndt et al. 2015; Wiemann et al. 2009; Linnemannstöns et al. 2002). Regarding the growing potential of industrial markets, where bikaverin could be applied, confirmation of the reliability of the previously reported metabolic route and its regulation is of great importance. To address this question, we provided in this study strong proofs of: (i) the consistency of the metabolic pathway for bikaverin synthesis in a *Fusarium* specie different from the laboratory model used so far, (ii) a more complete panel of the intermediates involved in the pathway, which had never been reported to that extent to our knowledge, and (iii) consistency in some regulatory patterns enabling more rational future strategies regarding optimization of fermentation conditions.

### Putative metabolic pathway for the bioproduction of bikaverin and intermediates thereof in *F. oxysporum* LCP531

If bikaverin itself has already been previously reported in some *Fusarium oxysporum* strains, its production ability and versatility amongst subspecies suggest that different metabolic pathways and/or regulatory patterns are involved. The above results confirmed that *F.* *oxysporum* LCP531 strain, when grown under the experimental conditions tested in this study, was able to produce preferably bikaverin **1** and its various intermediates (e.g., norbikaverin **2**, oxo-pre-bikaverin **3,** me-oxo-pre-bikaverin **4**, dinor-bikaverin **5** and pre-bikaverin **6**) with confirmed structures against the previously reported fusarubin-like pigments such as the 8-*O*-methyl nectriafurone (yellow pigment) or 8-*O*-methylfusarubin (red pigment) produced by *F. fujikuroi* (Studt et al. 2012), but not observed here. Indeed, none of the early intermediates of the bikaverin pathway have been reported from cultures of *F.* *oxysporum* under laboratory conditions until now. Such conclusions were first surprising, as the occurrence of the PGL gene cluster (consisting of homologs of the adjacent genes PGL1, PGL2 and PGL3) in the genome of *F.* *oxysporum* has been previously reported in *F. oxysporum* (Ma et al. 2010, 2013; Hansen et al. 2012; Brown et al. 2012), and was demonstrated to be involved in the synthesis of fusarubin-like naphthoquinone pigments by *F. fujikuroi* (Studt et al. 2012). Thus, it was assumed that the cultivation conditions used in this study have influenced the activity of the gene cluster regulating the route of naphthoquinones synthesis, consequently resulting in the final generation of pigmented components with slightly different chemical features or in the production of different colored intermediates of the pathway encoded by these genes.

**Fig. 6:**
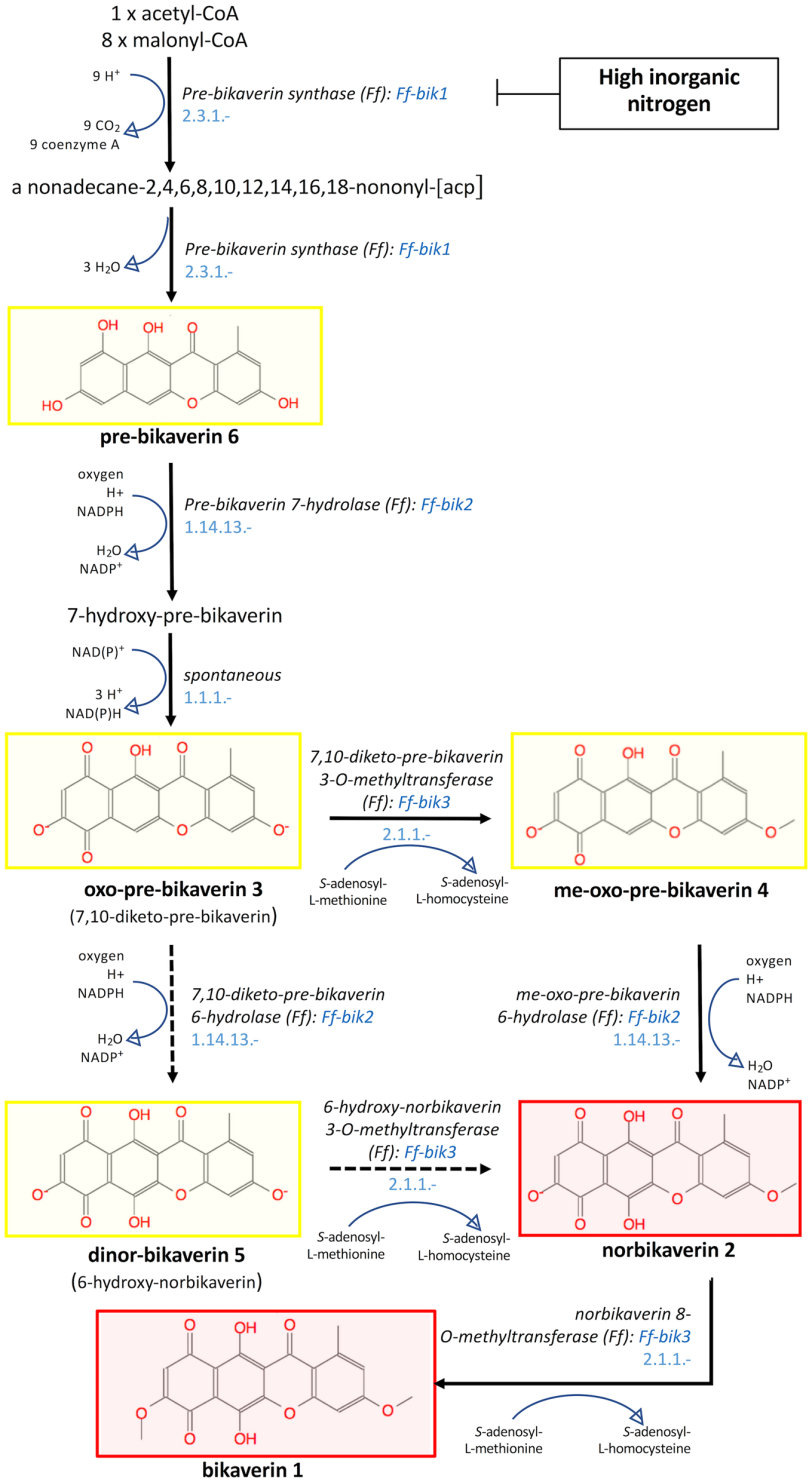
Putative metabolic pathway for the bioproduction of bikaverin **1** and intermediates thereof in *F. oxysporum* LCP531 based on both the identified compounds profiles produced in either intracellular or extracellular extracts and the previously reported model for bikaverin biosynthesis in *F. fujikuroi* (Arndt et al. 2015; Caspi et al. 2018). Numbers are referring to the compounds characterized in Figs. 2 and 3, and colors correspond to the shades of the pigments isolated in this study. Major pigments are highlighted in bold (bikaverin **1**, norbikaverin **2**, oxo-pre-bikaverin **3**, me-oxo-pre-bikaverin **4**, dinor-bikaverin **5**, and pre-bikaverin **6**). Pre-bikaverin was first described by Ma et al. (2007)

As proposed by Arndt and co-workers (2015) in *F. fujikuroi* model, the norbikaverin **2**, oxo-pre-bikaverin **3,** me-oxo-pre-bikaverin **4**, dinor-bikaverin **5** and pre-bikaverin **6** identified in this study, could be considered as putative intermediates in the putative biosynthetic pathway for bikaverin **1** synthesis in *F.* *oxysporum* (Arndt et al. 2015; review in: Caro et al. 2017). From a pathway point of view, it is reasonable to presume that the genes required for biosynthesis of these putative bikaverin ‘intermediates’ would be located in *F.* *oxysporum* genome within the same BIK gene cluster formed in *F.* *fujikuroi* by the responsible non-reducing PKS-encoding gene BIK1 (BIKaverin polyketide synthase), previously known as PKS4 (FFUJ_06742), and the five adjacent genes BIK2-BIK6 (Arndt et al. 2015; review in: Caro et al. 2017). These latter five genes encode a putative FAD-dependent monooxygenase (BIK2; FFUJ_06743), a putative *O*-methyltransferase (BIK3, FFUJ_06744), a putative NmrA-like transcriptional regulator (BIK4, FFUJ_06745), a putative Zn(II)_2_Cys_6_ fungal-type transcription factor (BIK5, FFUJ_06746) and a putative major facilitator superfamily (MFS) transporter (BIK6, FFUJ_06747) (Linnemannstöns et al. 2002; Wiemann et al. 2009; Schumacher et al. 2013). Deletion of any of these genes was reported to lead to reduction or complete loss of pigment production. Through the combination of genetic engineering and HPLC-HRMS analysis, the bikaverin biosynthetic pathway in F. *fujikuroi* was recently proposed by Arndt et al. (2015). Pre-bikaverin **6** has been recognized as the first bikaverin biosynthetic pathway intermediate and product of the gene BIK1: the condensation of 8 malonyl-CoA molecules and one acetyl-CoA molecule, catalyzed by the biosynthetic gene BIK1, resulted in the formation of the compound pre-bikaverin **6** in *F.* *fujikuroi*. Then, a monoxygenase (BIK2) oxidizes pre-bikaverin to form oxo-pre-bikaverin **3**, which is further methylated to me-oxo-pre-bikaverin **4** by a putative O-methyltransferase (BIK3). This intermediate is than hydroxylated by BIK2 to yield norbikaverin **2**, which is finally methylated to bikaverin **1** by BIK3 in *F.* *fujikuroi* (Arndt et al. 2015). The intermediate oxo-pre-bikaverin **3** could be also hydroxylated by BIK2 to yield dinor-bikaverin **5,** which is further methylated by BIK3 to norbikaverin **2** (Arndt et al. 2015; review in: Caro et al. 2017). The fungus *F.* *oxysporum* has been reported to possess this BIK gene cluster in its genome (Ma et al. 2010, 2013). Thus, the chemical structures and a putative metabolic pathway for the bioproduction of bikaverin **1** and intermediates thereof in *Fusarium oxysporum,* based on the secondary metabolites isolated in this study and the previously reported model for bikaverin biosynthesis in *F. fujikuroi* (Arndt et al. 2015; Caspi et al. 2018*)* was proposed and described in Fig. 6.

However, one question remains: if the occurrence of the genetic tools for bikaverin bioproduction in *Fusarium oxysporum* have been confirmed, how come bikaverin and all of its intermediates were not constitutively detected at same levels under our culture conditions tested? We reported hereby twice as much bikaverin **1**, norbikaverin **2** as well as higher concentrations of the earlier intermediates oxo-pre-bikaverin **3**, me-oxo-pre-bikaverin **4**, dinor-bikaverin **5** and pre-bikaverin **6** in mycelia extracts obtained from submerged cultures on DMD against PDB. Such results are supported by literature, where bikaverin production was shown as being strongly triggered by the nitrogen starvation, a low N/C ratio and acidic pH (Bu’lock et al. 1974; Giordano et al. 1999; Bell et al. 2003). Indeed, the DMD medium contained only ammonium sulfate (1 g/L) as nitrogen source and 30 g L^−1^ of glucose, resulting in a low overall N/C ratio (*ab*. 0.03), therefore yielding optimal conditions for bikaverin production. On the other hand, PDB hold all the nutrients that have been reported to inhibit bikaverin synthesis and in particular its high nitrogen content and its amino acids profile as glutamine was reported with inhibitory effect (Wiemann et al. 2009). Based on these observations also reported for the regulation of bikaverin pathway in *Fusarium fujikuroi* (Limón et al. 2010; Arndt et al. 2015), it is reasonable to assume that similar regulation mechanisms via nitrogen depletion are involved. While deletion of BIK1 resulted in complete absence of pigmentation in mutants of *F. fujikuroi*, according to Wiemann et al. (2009) and Rodríguez-Ortiz et al. (2010), BIK1 upregulation after nitrogen starvation was reported as the fastest responding gene from the bikaverin cluster, thus strongly supported our assumption that bikaverin biosynthesis was greatly favored in DMD medium and inhibited in PDB. Overall, these observations fully support our proposed bikaverin pathway in *F.* *oxysporum* (Fig. 6) with similar regulatory signals as reported in *F.* *fujikuroi*.

Paradoxically, many efforts were performed for the isolation, structural and biological characterization (Escamilla-Silva et al. 2001; Chelkowski et al. 1992; Balan et al. 1970; Bu’lock et al. 1974, Bekaert et al. 1992), but little was carried out to generate high titers of bikaverin and/or its derivatives. Amongst the few studies that investigated medium composition regarding bikaverin and other biocompounds production, Lale and Gadre (2016) confirmed the need of low N/C ratio and showed that undigested nitrogen sources form defatted plants meals significantly enhanced bikaverin titers. Therefore, to further improve bikaverin and its derivatives, variations of the N/C ratios and sources of the DMD medium should be further performed in order to favor the production of bikaverin and related-compounds only.

### Occurrence of side bioproduction of beauvericin in *F. oxysporum* LCP531 only under PDB cultivation

Additionally, we also identified in extracellular extracts of *F. oxysporum* the well-known predominant polyketidic mycotoxin beauvericin (Additional file 1: Figs. S12b; S13-B; Table 3), largely previously reported in *F.* *oxysporum* cultures. Interestingly, in our study, BEA was detected as the main extrolite produced exclusively when the fungus was grown in PDB submerged culture exposed to either light or darkness, while it was also observed in mycelial extract in other studies cultivating *F. oxyporum* in solid culture, like PDA agar plates (Zhan et al. 2007; Combès et al. 2012). According to its reported metabolic pathway in several *Fusarium* species, beauvericin is an intermediate of the valine degradation pathway (ESYN1 pathway) (Liuzzi et al. 2017) (Additional file 1: Fig. S12b). It is then coherent to isolate higher concentrations of such compound when the fungus was grown on amino acids enriched media (PDA or PDB contains yeast extracts and complex nitrogen sources from potato broth), while the strain was unable to produce beauvericin in neither intra- nor extracellular extracts of *F.* *oxysporum* LCP531 when grown on minimal medium (exposed to either light or darkness) not containing any complex nitrogen sources, and in particular amino acid sources. Furthermore, the gene clusters involved in the synthesis of BEA, consisting of the non-ribosomal peptide synthetase gene NRPS22 encoding the BEA synthases (BEA1-3), was described in the genome of *F.* *oxysporum* (Hansen et al. 2012; Niehaus et al. 2016), confirming the strain ability to produce such metabolite.

Such observation provides key information on ways to further enhance or limit specific mycotoxins occurrence. Although, BEA can be initially seen as an undesirable feature to be found in products intended to human consumption, it has been proven to be a promising bioactive agent for both agricultural and medical applications (Wang and Xu 2012; Liuzzi et al. 2017; Wu et al. 2018). Indeed, one of the main valuable property of beauvericin is it cytotoxicity, due to its acyl-CoA transferase inhibitory effect, with a focus on cholesterol transferase, consequently reducing the membrane plasticity and integrity of cells, and therefore favoring their decay (Liuzzi et al. 2017). Thus, the high production of this mycotoxin obtained here in extracellular extracts in PDB can be of great interest for progress in novel anticancer drugs development. Furthermore, the generous occurrence of BEA in a medium containing complex nitrogen source (potato starch) and glucose confirmed the conclusions previously reported by Wang and Xu (2012), which stated that simultaneous presence of glucose and peptone under medium pH and temperature conditions were favoring parameters for BEA biosynthesis. Nevertheless, the real biotechnological potential of BEA and mycotoxins alike would need more investigation into its bioproduction and regulation metabolic systems.

Here, we confirmed: (i) the ability of *F. oxysporum* LCP531 to produce BEA in submerged culture for potent biotechnological interest and application; (ii) the specific biosynthesis of BEA when complex peptidic-derivatives (casamino acids, yeast extracts, peptone, tryptone or potato broth) are present in the culture broth, and (iii) inversely, the complete absence of BEA in non-containing amino acids minimal medium, providing ways for production monitoring.

Lastly, our findings based on HPLC–DAD and UHPLC-HRMS analyses performed on both the IC and EC extracts of *F. oxysporum* confirmed the absence of other well-known mycotoxins (aurofusarin, zearalenone, fumonisins, trichothecenes (T2-toxin, nivalenol and deoxynivalenol), fusarin C and fusarielins) of *Fusarium* species with health consequences to humans and animals. This was consistent with the observation that none of the gene clusters involved in the biosynthesis of these well-known mycotoxins was described in the genome of *F.* *oxysporum* (Kim et al. 2005; Frandsen et al. 2006; Gaffoor and Trail 2006; Lysøe et al. 2006; Proctor et al. 2008; Song et al. 2004; Díaz-Sánchez et al. 2012; Sørensen et al. 2012).

### Description of side bioproduction of ergosterol in *F. oxysporum* LCP531 only in intracellular extracts

In addition to pigmented compounds, we reported the production of an ergosterol-derivate **10** only in intracellular extracts from either culture on DMD or PDB (Figs. 2 and 3; Table 2; Additional file 1: Fig. S12a). This result is in complete sense with the fact that ergosterol is one of the major constituents of fungal cell membrane (Slominski et al. 2005; Alcazar-Fuoli et al. 2008; Dupont et al. 2012; Dame et al. 2016), and is involved in survival mechanisms by enhancing the resistance of cell membrane against destructive oxidation of the membrane phospholipids. If the biosynthetic pathway of ergosterol has been well-characterized in *Saccharomyces cerevisiae*, in *Chlamydomonas reinhardtii* and other green algaes, little information is available for the fungal pathway (Da Silva Ferreira et al. 2005; Alcazar-Fuoli et al. 2008; Zhao et al. 2010). Other studies investigated the relationship between ergosterol and the production of common polyketidic mycotoxins (i.e. fumonisin B_1_, zearalenone and deoxynivalenol) of *Fusarium* spp., with no positive correlation that could be concluded (Stanisz et al. 2015), suggesting that ergosterol-derivates are being produced from a different pathway than common mycotoxins, and therefore can be obtained independently, if desired.

Ergosterol is also long-known as provitamine D that was used as antirachitic treatment, and more recently as potent supplementation in feed and food industries (Marova et al. 2010). To date, only few reports have investigated the production of ergosterol (ergosta-5,7,22-trien-3β-ol) derivatives from *Fusarium* species for pharmaceutical applications, such as those isolated from *F.* *proliferatum* that showed potent biological properties in medical field (Fangkrathok et al. 2013; Dame et al. 2016). Therefore, the hereby description of ergosterol-like compound as major secondary metabolites from intracellular extracts of *F. oxysporum* LCP531 opens ways to investigate potential new biological activities (i.e. cytotoxic, antitumor, immunostimulating, antifungal and antimicrobial drugs) (Torres et al. 2017).

As far as for other type of secondary metabolites, being pigments and/or mycotoxins, it is easy to predict that fungal strains have plenty of other biomolecules to reveal with as many applications, or to rediscover for novel usages. For instance, the well-known melanin-like pigments (polyketides) are gaining new interest in biomaterials design, and more specifically in the field of next-generation of semi-conductors and biopolymers (Mostert et al. 2012; Di Mauro et al. 2017, Markham et al. 2018). Despite the complex regulation of production of such secondary metabolites by a combined effect of environmental and epigenetic factors, the genetic and enzymatic toolkit remain crucial. We previously demonstrated the significant impact of the growth conditions on the biosynthesis of specific pigments such as wild-type purple naphthoquinone pigments produced by *F. oxysporum* (Lebeau et al. 2019), as well as for the production of bikaverin, ergosterol, and beauvericin with bioactivities. Moreover, other studies also confirmed the significant impact of the environment conditions and nutrients profiles on bioproduction of specific biomolecules, such as the availability and nature of nitrogen/carbon sources for favoring bikaverin generation (Garbayo et al. 2003). Here, the further investigation on available literature combined to genome blast and analysis of secondary metabolic spectral profiles obtained under the various growth conditions tested in our study led us to the confirmation of occurrence across two different *Fusarium* sp of the above described biosynthetic pathways of bikaverins and its derivates in wild *F. oxysporum* LCP531. Knowing more about their metabolic pathways and confirming their occurrence and regulation across strain species provides key elements to help optimizing more efficiently culture conditions, as well as identifying genetic engineering to be performed to further improve or create new enzymatic functionalities (Klaus and Grininger 2018) for the bioproduction of specific biocompounds for industrial, agricultural and pharmaceutical applications. To this date, there is still a strong need of more detailed genomic and metabolomic databases to further complete our assumptions. The next step would require a combination of experimental knock-in/knock-out analyses to draw a steady map of available biosynthetic routes and favoring environmental conditions for the specific generation of potential highly valuable biocompounds, and potentially pave the way to tunable bioproduction of one compound to another in a more diverse panel of *Fusarium* strains.

## Supplementary information

**Additional file 1.** Additional figures.

## Data Availability

All data generated or analysed during this study are included in this published article (and its additional information file).

## Abbreviations

BEA: beauvericin
BIK: bikaverin polyketide synthase
DMD: defined minimal dextrose broth
EAC: Ehrlich ascites carcinoma
EC: extracellular extracts
EtOH: ethanol
HPLC-DAD: high-performance liquid-chromatography combined with photodiode array-detection
HRMS: high-resolution mass spectrometry
IC: intracellular extracts
MeOH: methanol
meqv.: milli-equivalents
PDB: potato dextrose broth
UV: ultra-violet
