# Supplementary material for: Putative metabolic pathway for the bioproduction of bikaverin and intermediates thereof in the wild Fusarium oxysporum LCP531 strain
Source: AMB Express. 2019 Nov 20;9:186. doi: 10.1186/s13568-019-0912-4 (PMC6868082; doi:10.1186/s13568-019-0912-4)
Supplement: Supplementary file 1 — Additional file 1. Additional figures. [file 13568_2019_912_MOESM1_ESM.pdf]

AMB Express

Original article

Putative metabolic pathway for the bioproduction of bikaverin and intermediates thereof in the wild *Fusarium oxysporum* LCP531 strain

Juliana Lebeau [1], Thomas Petit [1,2], Laurent Dufossé [1] and Yanis Caro [1,2,*]

[1] Laboratoire de Chimie des Substances Naturelles et des Sciences des Aliments (LCSNSA), Université de La Réunion, 15 avenue René Cassin, CS 92003, F-97744, Saint-Denis, Réunion, France ;
[2] Département Hygiène Sécurité Environnement (HSE), IUT La Réunion, Université de La Réunion, 40 avenue de Soweto, BP 373, F-97455, Saint-Pierre, Réunion, France.

* Corresponding author: Email: yanis.caro@univ-reunion.fr; Tel.: +262-262-483-361

Additional file 1

Contents

Figure S1. Chemical structure and mass spectrum of bikaverin **1**

Figure S2. Chemical structure and mass spectrum of norbikaverin **2**

Figure S3. Chemical structure and mass spectrum of oxo-pre-bikaverin **3**

Figure S4. Chemical structure and mass spectrum of me-oxo-pre-bikaverin **4**

Figure S5. Chemical structure and mass spectrum of dinor-bikaverin **5**

Figure S6. Chemical structure and mass spectrum of pre-bikaverin **6**

Figure S7. Absorption spectrum of the compound **7a** not tentatively identified.

Figure S8. Absorption spectrum of the compound **7b** not tentatively identified.

Figure S9. Absorption spectrum of the compound **7c** not tentatively identified.

Figure S10. Absorption spectrum of the compound **8** not tentatively identified.

Figure S11. Absorption spectrum of the compound **9** not tentatively identified.

Figure S12. Chemical structures and biosynthesis pathways of (a) ergosterol and (b) beauvericin in fungi. The pathway of ergosterol was based on the bioproduction route of *Saccharomyces cerevisiae* and reported as similar in fungi (adapted from Dupont et al. 2012; Alcazar-Fuoli et al. 2008). The beauvericin biosynthesis pathway was adapted from Wang and Xu (2012).

Figure S13. HPLC-DAD chromatograms of the extracellular aqueous ethanolic* extract (EC) obtained from the lyophilized 7-day-old fermentation broth (culture supernatant) of *F. oxysporum* LCP531 culture either (A) on define minimal dextrose broth (DMD), (B) or on potato dextrose broth (PDB).

Table S1. Composition of the two liquid media, Potato Dextrose Broth (PDB) and Defined Minimal Dextrose broth (DMD), used as culture medium and prepared using sterile distilled water according to Lebeau et al. (2017) for fungal cultures of *Fusarium oxysporum*.

Figure S1. Chemical structure and mass spectrum of bikaverin **1**

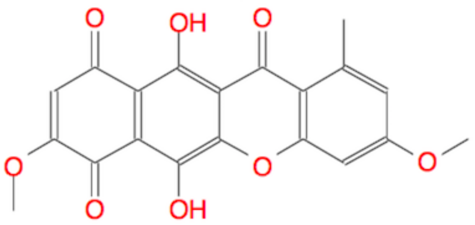

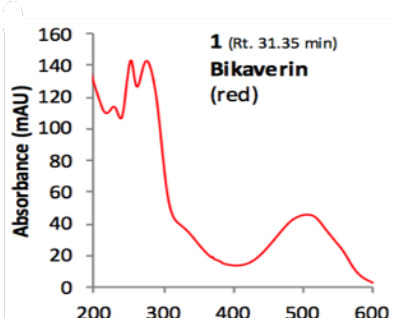

Absorption spectra: λ_{max} 195, 228, 253, 276, 336, 507 nm.

ESI-MS molecular ion: *m/z* 383.0292 in positive mode $[M+H]^+$

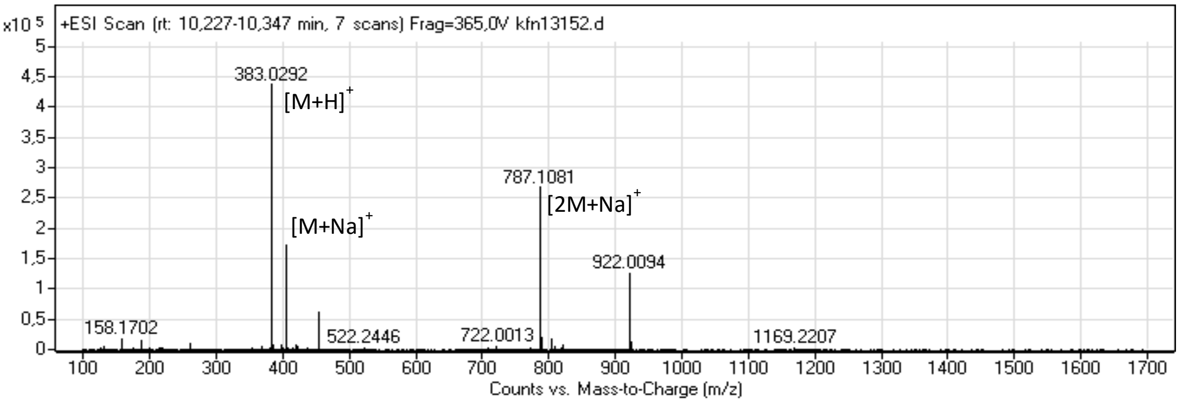

Figure S2. Chemical structure and mass spectrum of norbikaverin **2**

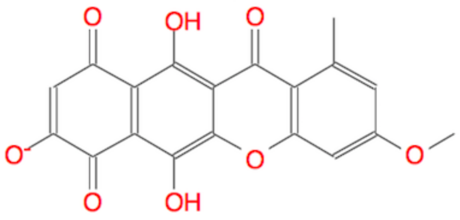

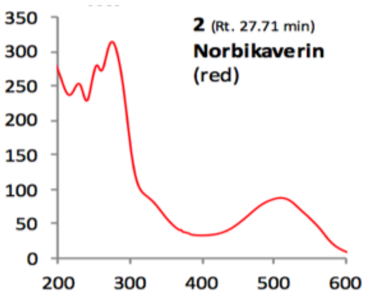

Absorption spectra: λ_{max} 196, 228, 254, 275, 336, 509 nm

ESI-MS molecular ion: *m/z* 369.0147 in positive mode $[M+H]^+$

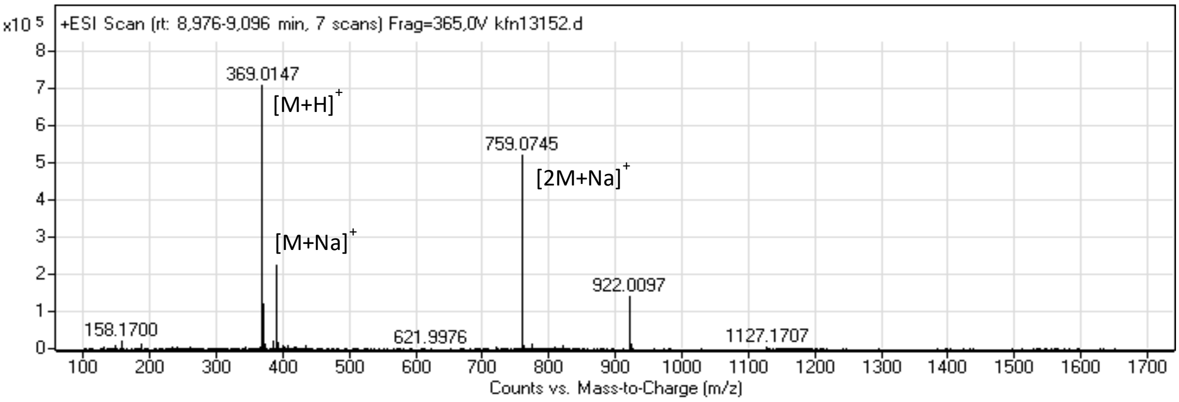

Figure S3. Chemical structure and mass spectrum of oxo-pre-bikaverin **3**

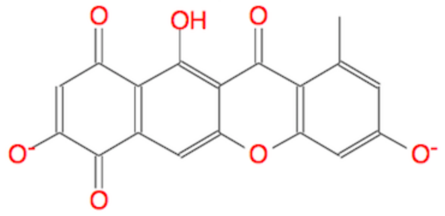

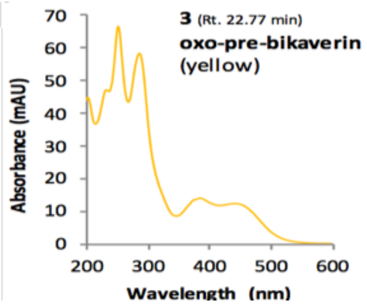

Absorption spectra: λ_{max} 200, 249, 285, 382, 441 nm.

ESI-MS molecular ion: *m/z* 339.0070 in positive mode $[M+H]^+$

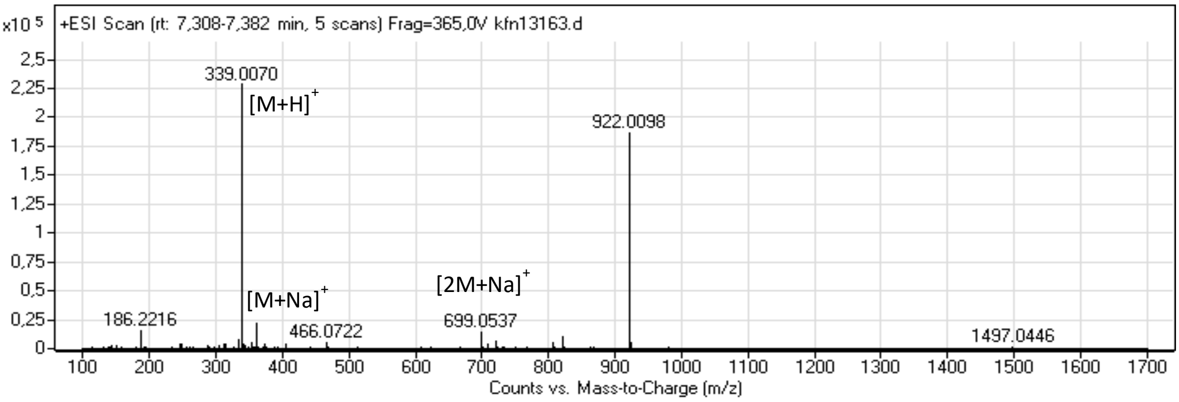

Figure S4. Chemical structure and mass spectrum of me-oxo-pre-bikaverin **4**

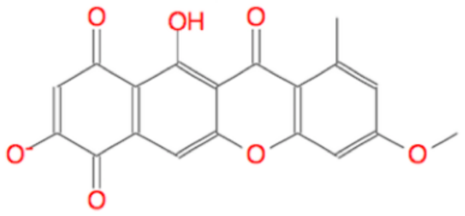

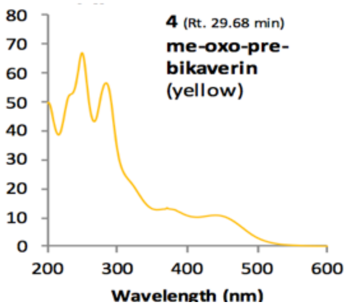

Absorption spectra: λ_{max} 200, 249, 284, 376, 440 nm.

ESI-MS molecular ion: *m/z* 353.0205 in positive mode $[M+H]^+$

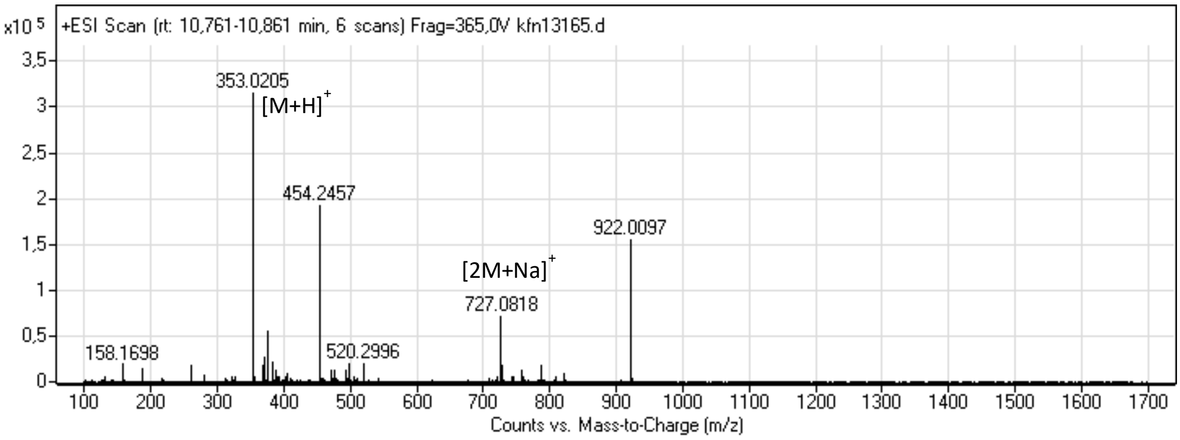

Figure S5. Chemical structure and mass spectrum of dinor-bikaverin **5**

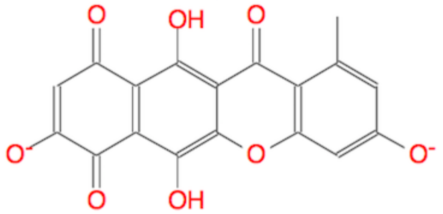

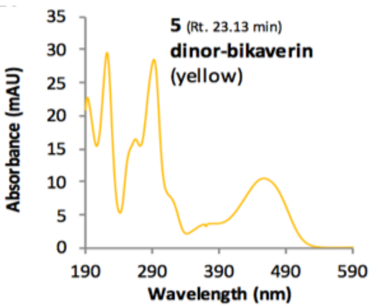

Absorption spectra: λ_{max} 193, 222, 265, 292, 375, 457 nm.

ESI-MS molecular ion: *m/z* 357.0520 in positive mode $[M+H]^+$

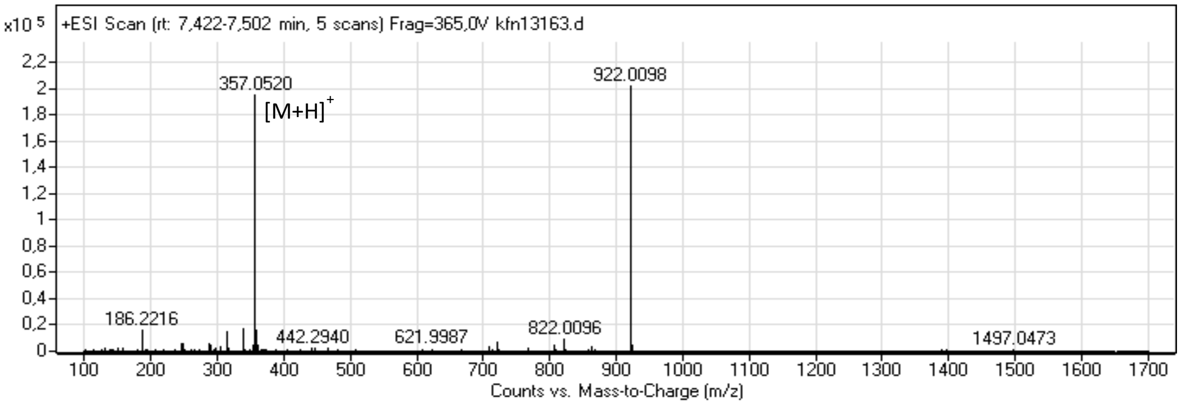

Figure S6. Chemical structure and mass spectrum of pre-bikaverin **6**

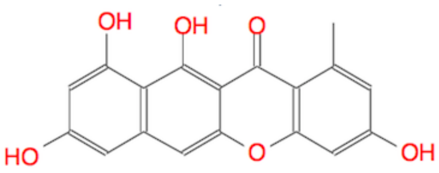

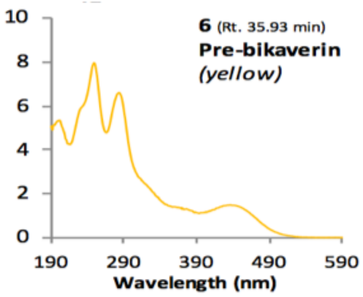

Absorption spectra: λ_{max} 202, 250, 284, 375, 438 nm.

ESI-MS molecular ion: *m/z* 323.1049 in positive mode $[M+H]^+$

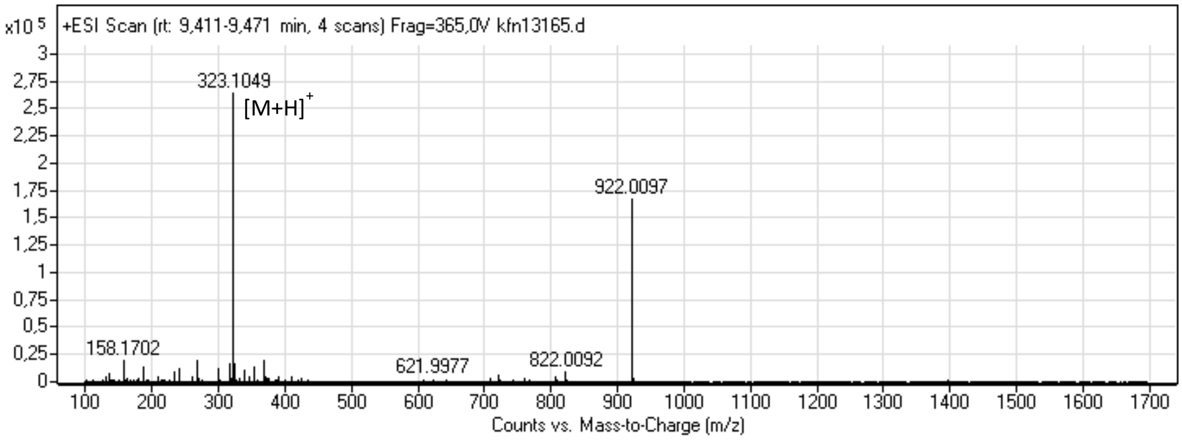

Figure S7. Absorption spectrum of the compound **7a** not tentatively identified. Absorption spectra: λ_{max} 200, 249, 284, 376, 437 nm.

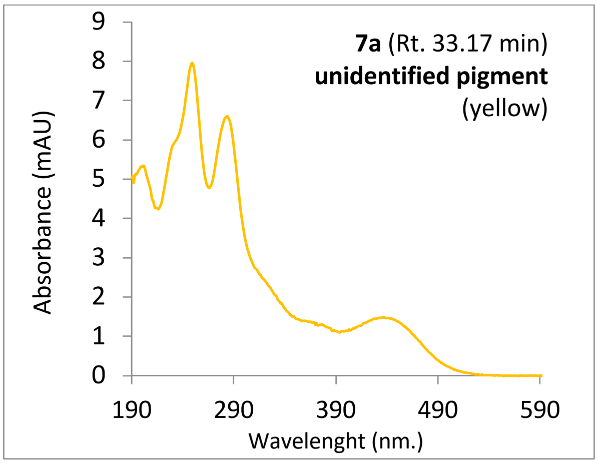

Figure S8. Absorption spectrum of the compound **7b** not tentatively identified. Absorption spectra: λ_{max} 191, 230, 262, 310, 496 and 523 nm.

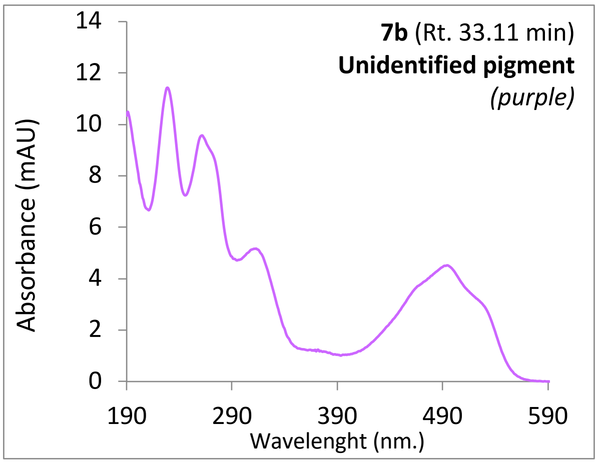

Figure S9. Absorption spectrum of the compound **7c** not tentatively identified. Absorption spectra: λ_{max} 230, 249, 276, 461, 491, 530 nm.

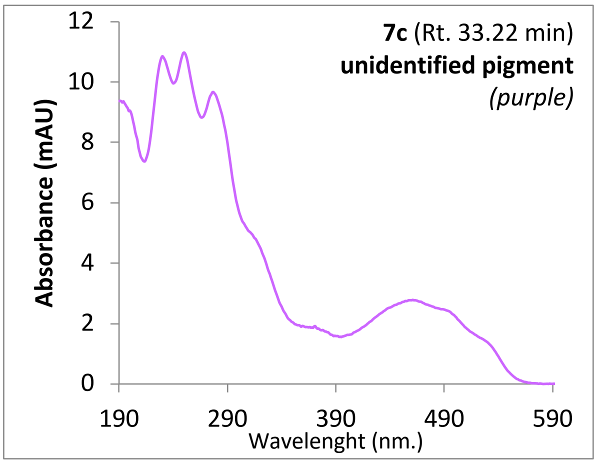

Figure S10. Absorption spectrum of the compound **8** not tentatively identified. Absorption spectra: λ_{max} 202, 228, 253, 277, 299, 332, 348, 430 nm.

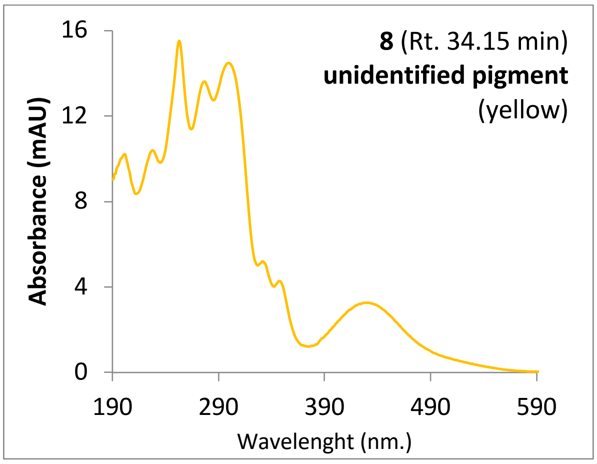

Figure S11. Absorption spectrum of the compound **9** not tentatively identified. Absorption spectra: λ_{max} 193, 243, 277, 324, 516, 549 nm.

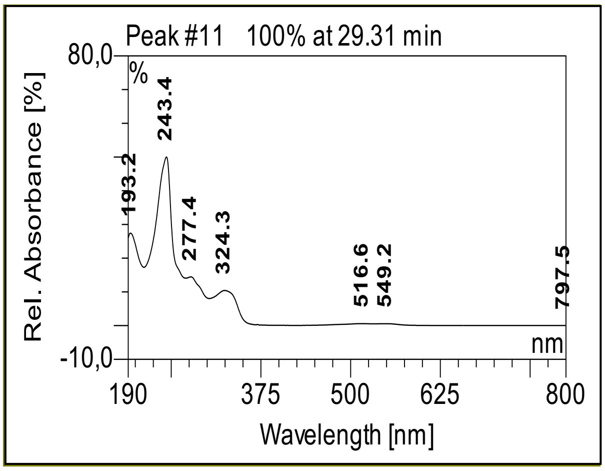

Figure S12. Chemical structures and biosynthesis pathways of (a) ergosterol and (b) beauvericin in fungi. The pathway of ergosterol was based on the bioproduction route of *Saccharomyces cerevisiae* and reported as similar in fungi (adapted from Dupont et al. 2012; Alcazar-Fuoli et al. 2008). The beauvericin biosynthesis pathway was adapted from Wang and Xu (2012).

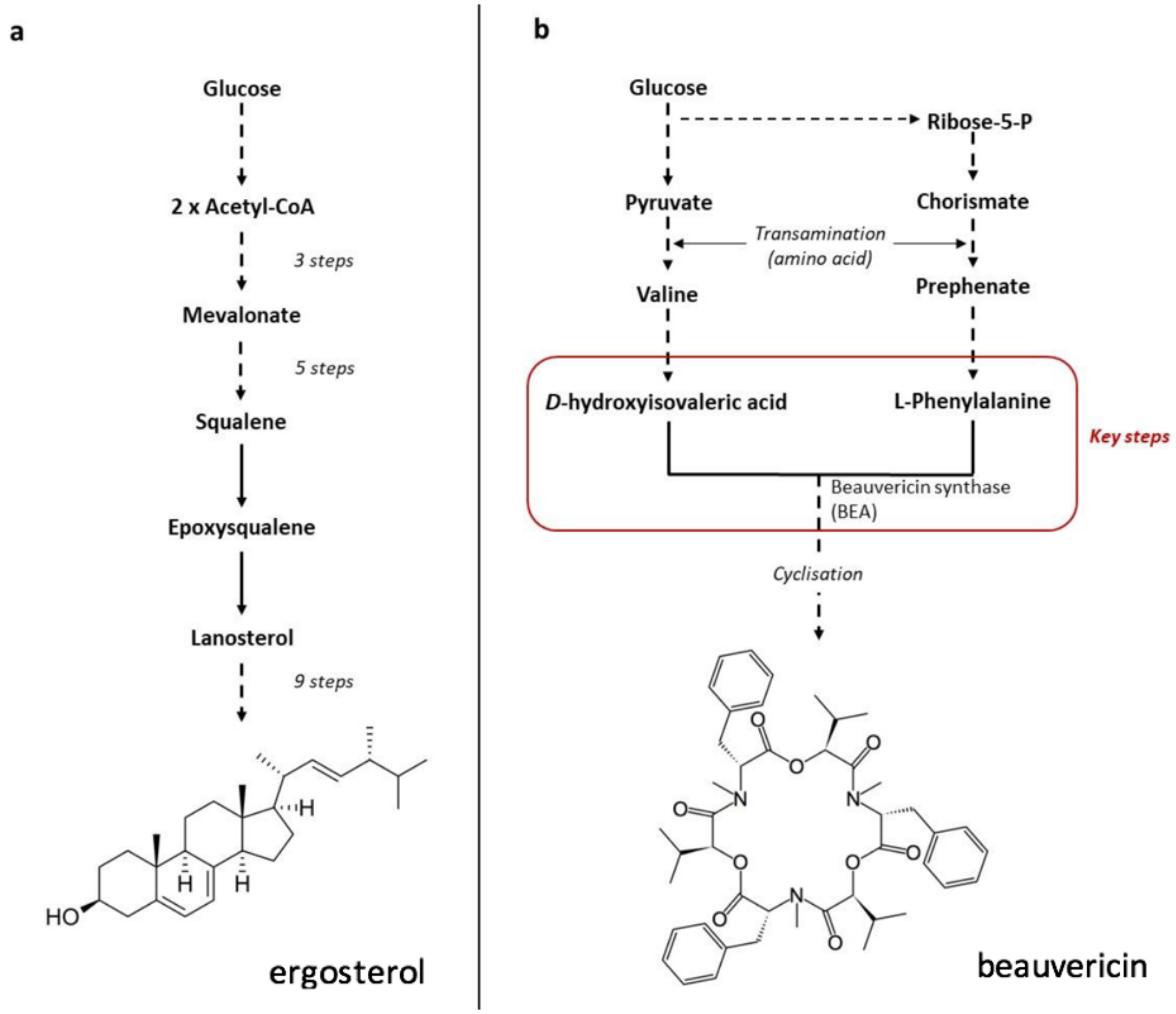

Figure S13. HPLC-DAD chromatograms of the extracellular aqueous ethanolic* extract (EC) obtained from the lyophilized 7-day-old fermentation broth (culture supernatant) of *F. oxysporum* LCP531 culture either **(A)** on define minimal dextrose broth (DMD), **(B)** or on potato dextrose broth (PDB).

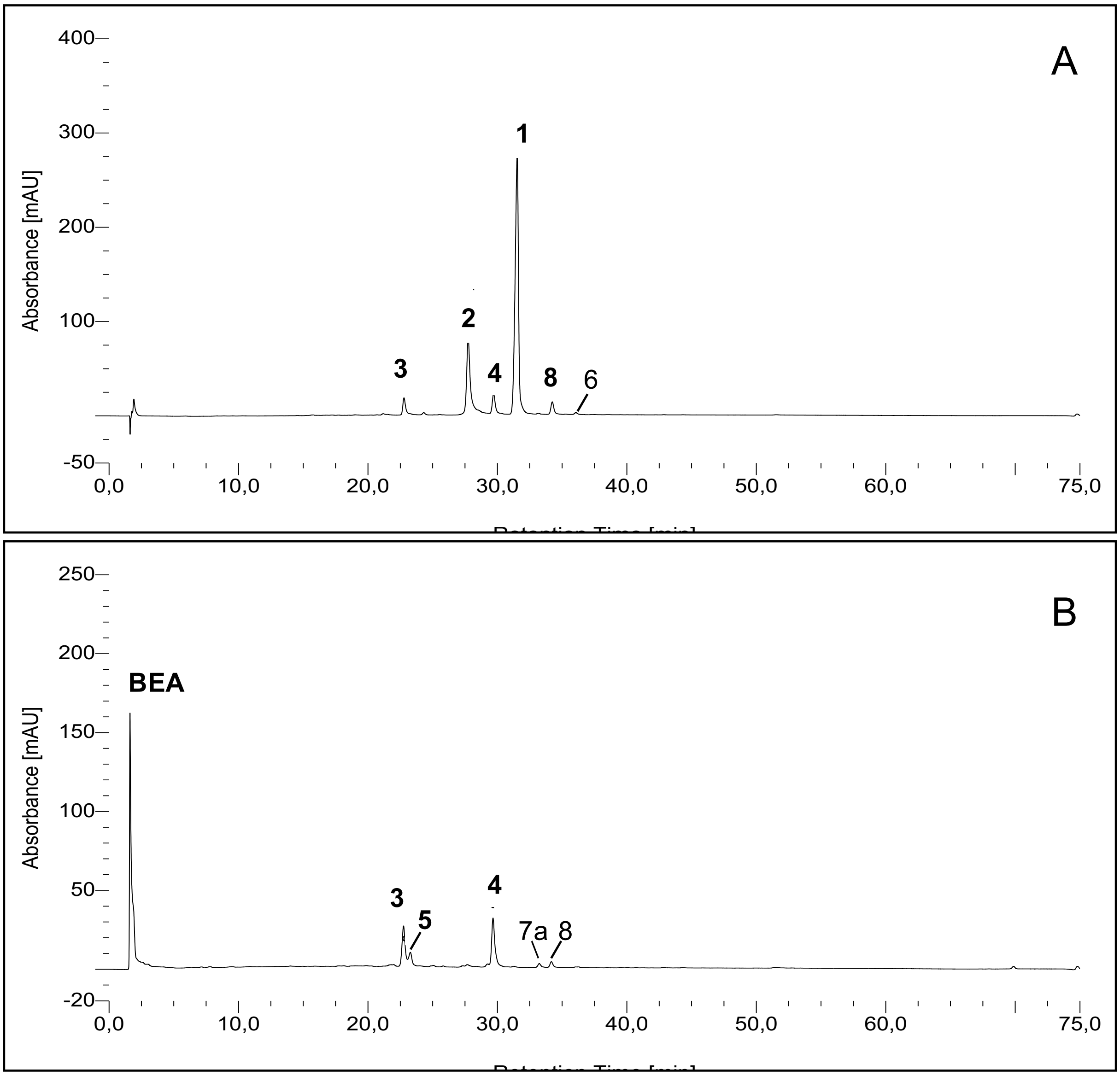

The 50% aqueous ethanolic extract was the most representative of the EC extracts obtained, due to the extrolites composition. Assignment of the bikaverin* ***1* *and possible intermediates (norbikaverin* ***2****, oxo-pre-bikaverin* ***3****, me-oxo-pre-bikaverin* ***4****, dinor-bikaverin* ***5****, pre-bikaverin* ***6****) and the beauvericin (BEA) were done by HRMS according to their mass to charge ratio. The minor extrolites, labelled as compounds 7a and 8 in the chromatograms were isolated but not identified.*

Table S1. Composition of the two liquid media, Potato Dextrose Broth (PDB) and Defined Minimal Dextrose broth (DMD), used as culture medium and prepared using sterile distilled water according to Lebeau et al. (2017) for fungal cultures of *Fusarium oxysporum.*

**Sources**		**PDB**	**DMD**
**Carbon source**	- simple:	sucrose (20g)	glucose (30g)
	- complex:	potato infusion (soluble starch) (4g)	-
**Nitrogen source**	- simple:	-	$(NH_4)_2SO_2$ (1g)
	- complex:	potato infusion (protein) (≅ 1g)	-
**Mineral salts**		potato infusion:	
		- P	- $MgSO_4^-$, $7H_2O$
		- Ca	- K_2HPO_4
		- Na	- KH_2PO_4
		- Mg (traces)	- $ZnSO_4$, H_2O
		- K (traces)	- $FeSO_4$, $7H_20$
		- Mn (traces)	- NaH_2PO_4
		- Fe (traces)	- $MnSO_4^-$, $2H_20$
		- Cu (traces)	- $CuSO_4^-$, $5H_2O$
		- Zn (traces)	
